# Supplementary material for: Pharmacological and genetic activation of cAMP synthesis disrupts cholesterol utilization in Mycobacterium tuberculosis
Source: PLoS Pathog. 2022 Feb 8;18(2):e1009862. doi: 10.1371/journal.ppat.1009862 (PMC8856561; doi:10.1371/journal.ppat.1009862)
Supplement: S1 Fig — (A) Inhibitory activity of V-59 against WT, the Rv1625c transposon mutant (Tn::rv1625c), and WT transformed with an overexpression plasmid expressing the rv1625c gene (2xrv1625c) in 7H12+cholesterol media. (B) Inhibitory activity of V-59 against WT, ΔRv1625c, and Compfull in 7H12+cholesterol media (left) or 7H12+cholesterol+acetate media (right). Data shown are representative, from one experiment with two technical replicates. Symbols are mean data points, and curves display nonlinear fit of dose-response. (C) Effect of V-59 on growth of Mtb in murine macrophages. Macrophages were infected at an MOI of 2 (left), 1 (middle), or 0.5 (right) and treated with V-59 (25 μM) or DMSO. Data for MOI of 2 are from three experiments, with two or three technical replicates each. Data for MOI of 1 and 0.5 are from one experiments with two or three technical replicates each. Data are shown as means ± SD. Each symbol indicates one replicate. (D) Effect of frontline antibiotics on WT and ∆Rv1625 in 7H12+cholesterol (INH, RIF, EMB) or in MES-buffered 7H9OADC+glycerol, pH 5.9 (PZA). Data shown are representative, from one experiment with two technical replicates. Symbols are mean data points, and curves display nonlinear fit of dose-response. (E) RNA-seq derived normalized counts of rv1625c reads in WT, ∆Rv1625c, and CompFull strains in 7H12+cholesterol media. (PDF) [file ppat.1009862.s001.pdf]

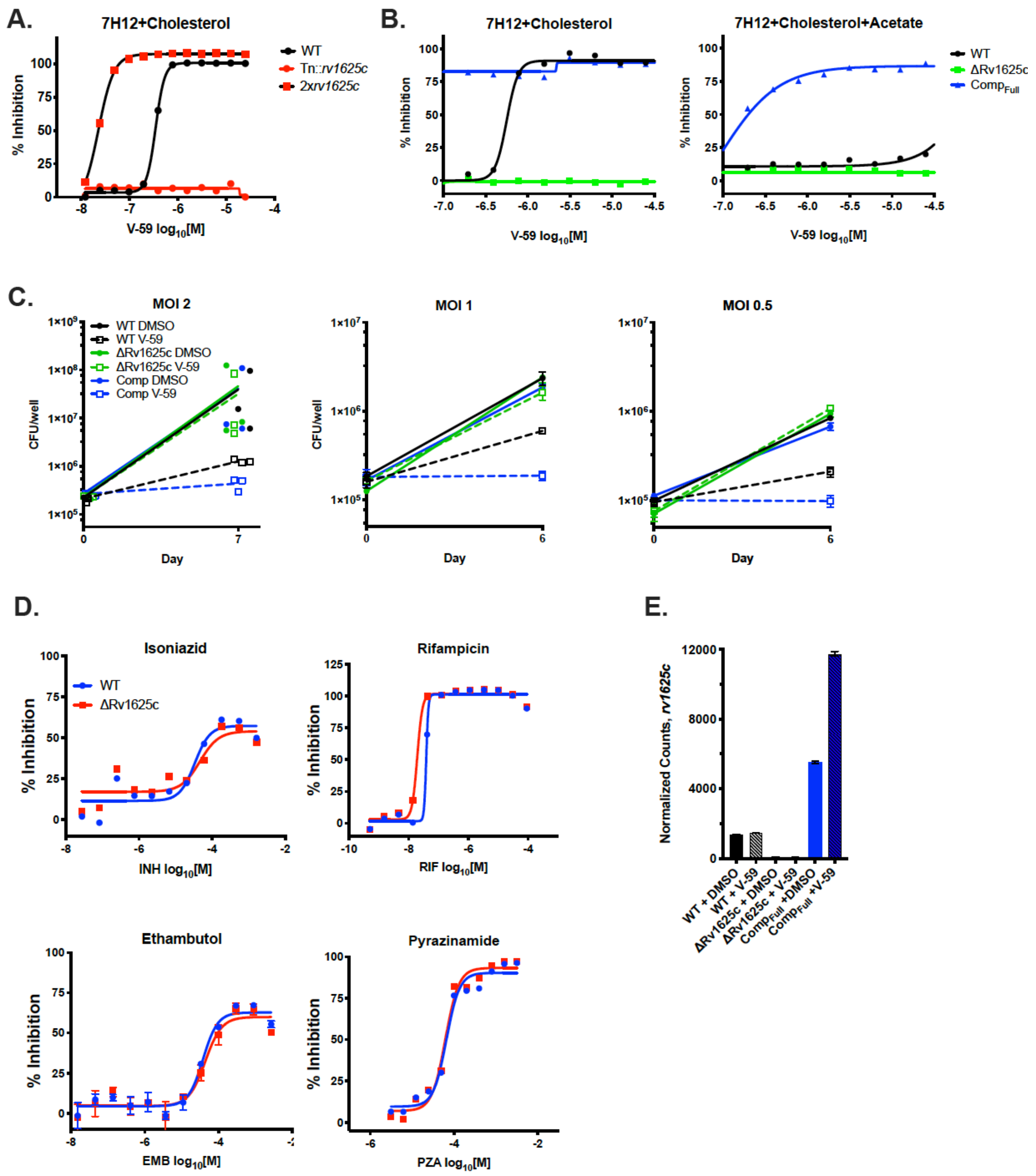

Fig. S1. The inhibitory activity of V-59 in cholesterol media is dependent on Rv1625c. (**A**) Inhibitory activity of V-59 against WT, the Rv1625c transposon mutant (Tn::*rv1625c*), and WT transformed with an overexpression plasmid expressing the *rv1625c* gene (2x*rv1625c*) in 7H12+cholesterol media. (**B**) Inhibitory activity of V-59 against WT, ΔRv1625c, and $Comp_{full}$ in 7H12+cholesterol media (left) or 7H12+cholesterol+acetate media (right). Data shown are representative, from one experiment with two technical replicates. Symbols are mean data points,

and curves display nonlinear fit of dose-response. (**C**) Effect of V-59 on growth of Mtb in murine macrophages. Macrophages were infected at an MOI of 2 (left), 1 (middle), or 0.5 (right) and treated with V-59 (25 μM) or DMSO. Data for MOI of 2 are from three experiments, with two or three technical replicates each. Data for MOI of 1 and 0.5 are from one experiments with two or three technical replicates each. Data are shown as means ± SD. Each symbol indicates one replicate. (**D**) Effect of frontline antibiotics on WT and ΔRv1625 in 7H12+cholesterol (INH, RIF, EMB) or in MES-buffered 7H9OADC+glycerol, pH 5.9 (PZA). Data shown are representative, from one experiment with two technical replicates. Symbols are mean data points, and curves display nonlinear fit of dose-response. (**E**) RNA-seq derived normalized counts of *rv1625c* reads in WT, ΔRv1625c, and $Comp_{Full}$ strains in 7H12+cholesterol media.
