# Supplementary material for: Pharmacological and genetic activation of cAMP synthesis disrupts cholesterol utilization in Mycobacterium tuberculosis
Source: PLoS Pathog. 2022 Feb 8;18(2):e1009862. doi: 10.1371/journal.ppat.1009862 (PMC8856561; doi:10.1371/journal.ppat.1009862)
Supplement: S2 Fig — (A) Inhibitory activity of mCLB073 or V-59 against spontaneous resistant mutants that were generated during culture with mCLB073. (B) Inhibitory activity of mCLB073 against WT or ΔRv1625c in 7H12+cholesterol media. Data shown are representative, from one experiment with two technical replicates. Symbols are mean data points, and curves display nonlinear fit of dose-response. (C) Impact of mCLB073 on cAMP production in WT Mtb. ELISA was used to quantify cAMP from lysed cells 24 hours after addition of mCLB073, or vehicle control (DMSO). Data is from one experiment, with two technical replicates. Data are shown as means ± SD. (PDF) [file ppat.1009862.s002.pdf]

A.

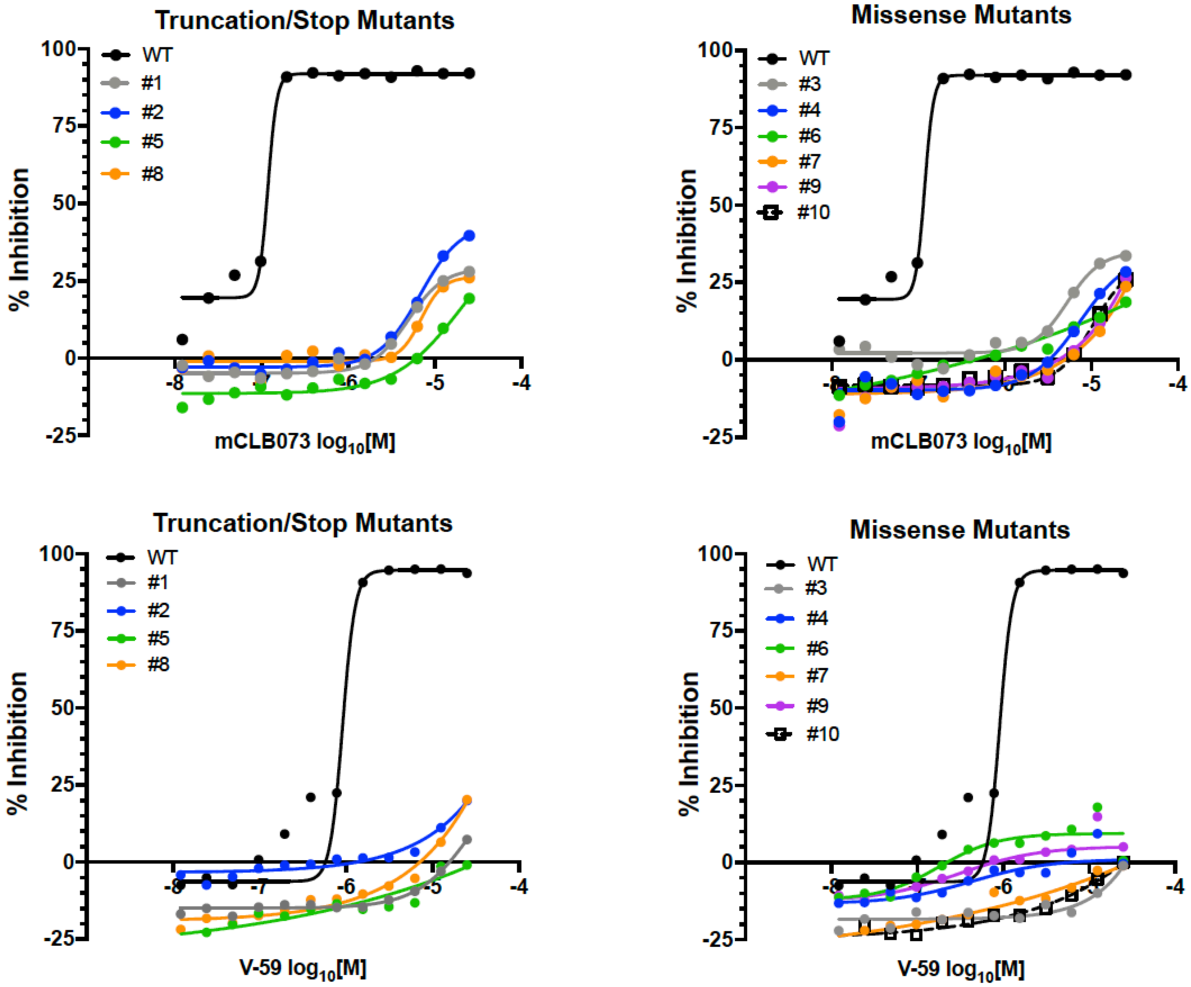
Truncation/Stop Mutants
% Inhibition
mCLB073 log10[M]
WT
#1
#2
#5
#8
Missense Mutants
% Inhibition
mCLB073 log10[M]
WT
#3
#4
#6
#7
#9
#10
Truncation/Stop Mutants
% Inhibition
V-59 log10[M]
WT
#1
#2
#5
#8
Missense Mutants
% Inhibition
V-59 log10[M]
WT
#3
#4
#6
#7
#9
#10

B.

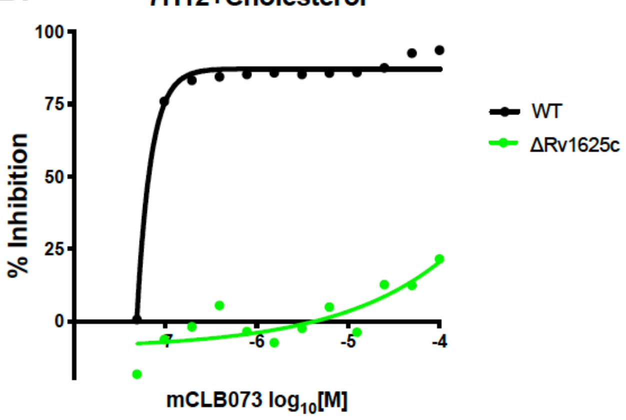
7H12+Cholesterol
% Inhibition
mCLB073 log10[M]
WT
ΔRv1625c

C.

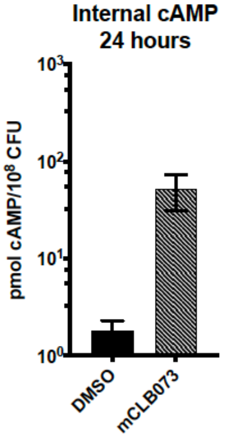
Internal cAMP
24 hours
pmol cAMP/10^8 CFU
DMSO
mCLB073

Fig. S2. The activity of mCLB073 is dependent on Rv1625c. (**A**) Inhibitory activity of mCLB073 or V-59 against spontaneous resistant mutants that were generated during culture with mCLB073. (**B**) Inhibitory activity of mCLB073 against WT or ΔRv1625c in 7H12+cholesterol media. Data shown are representative, from one experiment with two technical replicates. Symbols are mean data points, and curves display nonlinear fit of dose-response. (**C**) Impact of mCLB073 on cAMP production in WT Mtb. ELISA was used to quantify cAMP from lysed cells 24 hours after addition of mCLB073, or vehicle control (DMSO). Data is from one experiment, with two technical replicates. Data are shown as means ± SD.
