# Supplementary material for: Pharmacological and genetic activation of cAMP synthesis disrupts cholesterol utilization in Mycobacterium tuberculosis
Source: PLoS Pathog. 2022 Feb 8;18(2):e1009862. doi: 10.1371/journal.ppat.1009862 (PMC8856561; doi:10.1371/journal.ppat.1009862)
Supplement: S3 Fig — (A) Catabolic release of 14CO2 from [U-14C]-palmitate in media containing fatty acid. EtOH (control), V-59, or Atc were added to the TetOn-cAMP Mtb cultures 24 hours prior to the start of the experiment. Data are from one experiment with three technical replicates, normalized to OD and quantified relative to EtOH control. Data are shown as means ± SD (B) Catabolic release of 14CO2 from [4-14C]-cholesterol in media containing cholesterol and acetate. V-59 (10 μM) was added to the cultures once at the beginning of the experiments and DMSO was used as a vehicle control. Data are from one experiment with three technical replicates, normalized to OD and quantified relative to WT treated with DMSO. Data are shown as means ± SD. (C) Schematic illustrating the topology of the N-terminal transmembrane domain and essential residues of the C-terminal cyclase domain of Rv1625c (left). Schematics illustrating modified Rv1625c constructs used in these studies (center and right). (PDF) [file ppat.1009862.s003.pdf]

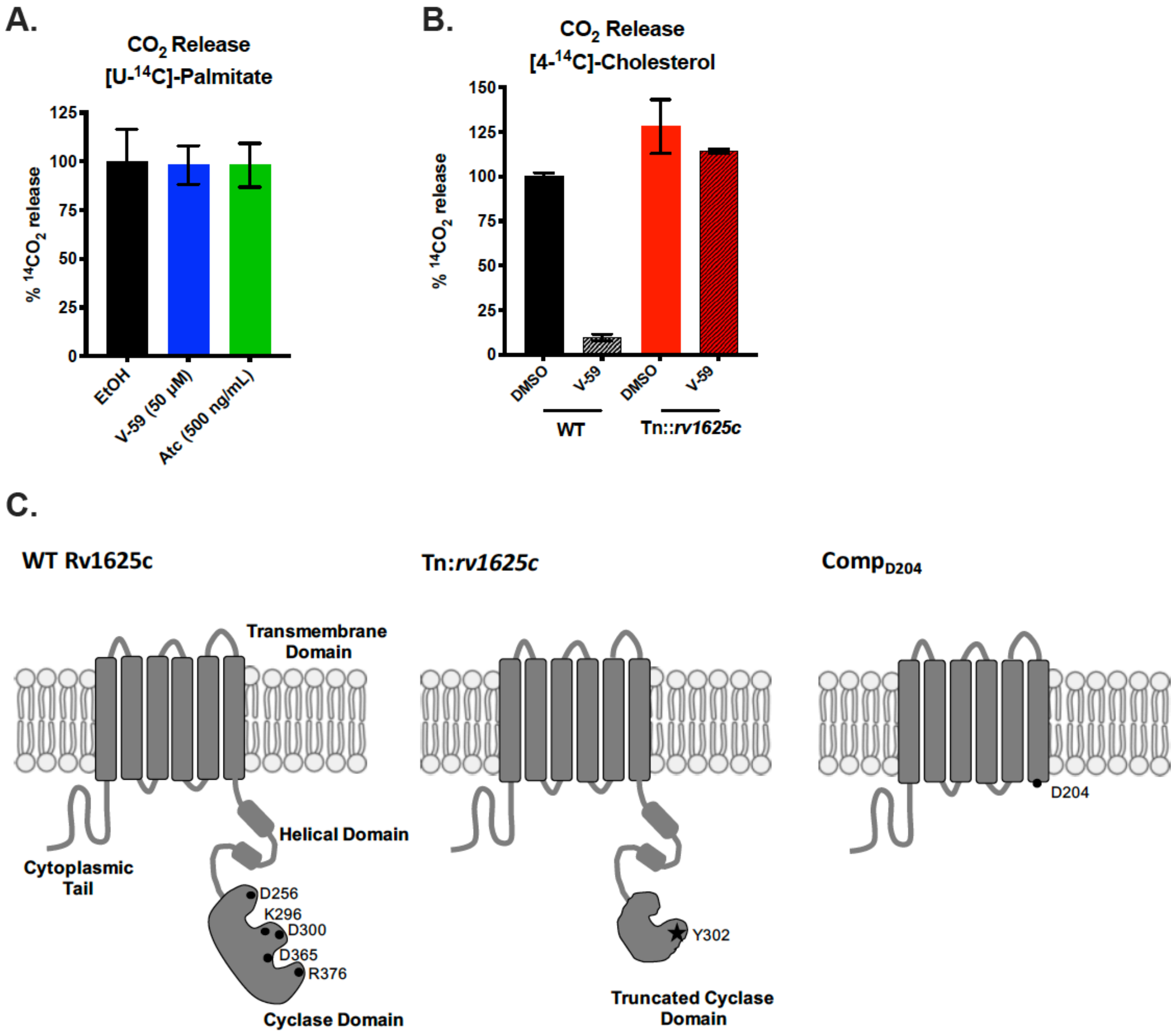

Fig. S3. An intact cyclase domain of Rv1625c is required for V-59 to inhibit cholesterol degradation. (**A**) Catabolic release of $^{14}CO_2$ from [U-^{14}C]-palmitate in media containing fatty acid. EtOH (control), V-59, or Atc were added to the TetOn-cAMP Mtb cultures 24 hours prior to the start of the experiment. Data are from one experiment with three technical replicates, normalized to OD and quantified relative to EtOH control. Data are shown as means ± SD (**B**) Catabolic release of $^{14}CO_2$ from [4-^{14}C]-cholesterol in media containing cholesterol and acetate. V-59 (10 μM) was added to the cultures once at the beginning of the experiments and DMSO was used as a vehicle control. Data are from one experiment with three technical replicates, normalized to OD and quantified relative to WT treated with DMSO. Data are shown as means ± SD. (**C**) Schematic illustrating the topology of the N-terminal transmembrane domain and essential residues of the C-terminal cyclase domain of Rv1625c (left). Schematics illustrating modified Rv1625c constructs used in these studies (center and right).
