# Supplementary material for: Pharmacological and genetic activation of cAMP synthesis disrupts cholesterol utilization in Mycobacterium tuberculosis
Source: PLoS Pathog. 2022 Feb 8;18(2):e1009862. doi: 10.1371/journal.ppat.1009862 (PMC8856561; doi:10.1371/journal.ppat.1009862)
Supplement: S4 Fig — (A) Schematic illustrating the domains of the native Rv1264 adenylyl cyclase (left) and the design of the TetOn-cAMP construct (right). The TetOn-cAMP construct contains the minimum-necessary cyclase domain of Rv1264, and lacks the pH-sensitive inhibitory domain of the native Rv1264 protein. Expression of the cyclase domain is under control of a TetOn promoter. Upon treatment with Atc, release of the tetracycline repressor (TetR) causes initiation of transcription of the Rv1264 catalytic domain. The C-terminal end of the cyclase domain is His-tagged to allow immunoblotting. (B) Immunoblots of bacterial lysates confirm that the TetOn constructs are expressed in the presence of Atc and not the vehicle control EtOH. The anti-His blot detects the Rv1264 cyclase domain and the anti-GroEL2 blot is the loading control. (C) Impact of inducing the TetOn-Rv1264D265A construct in media containing cholesterol and acetate. Cultures were treated with one dose of EtOH, V-59 (10 μΜ), or Atc at the indicated concentrations and samples were collected 24 hours later for ELISA. Data are displayed as total cAMP per 108 Mtb. Data are from two independent experiments with two technical replicates each, shown as means ± SD (**P < 0.01, One-way ANOVA with Dunnett’s multiple comparisons test). (D) Effect of inducing TetOn-Rv1264D265A on the growth of Mtb in 7H12+cholesterol media, monitored by serial OD measurements. EtOH, V-59 (10 μM), or Atc at the indicated concentrations were added initially and every three days for the duration of the experiment. Data are from one experiment with three technical replicates, shown as means ± SD. (E) Catabolic release of 14CO2 from [4-14C]-cholesterol in media containing cholesterol and acetate. The TetOn-Rv1264D265A strain was treated with EtOH, V-59 (10 μM), or Atc at the indicated concentrations overnight and again one hour prior to the beginning of the experiments. Data are from two independent experiments with three technical replicates each, normali [file ppat.1009862.s004.pdf]

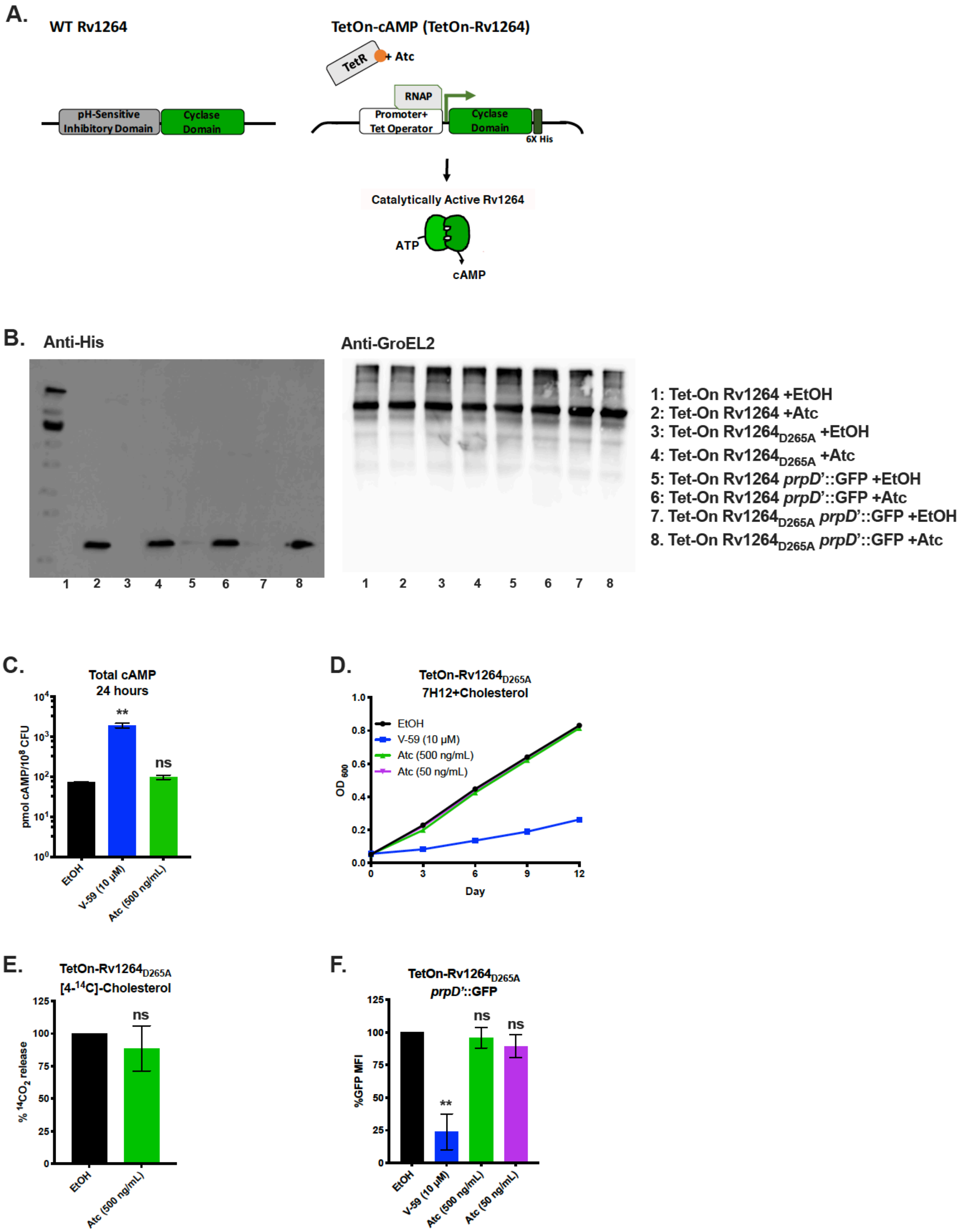
A.
WT Rv1264
pH-Sensitive Inhibitory Domain
Cyclase Domain
TetOn-cAMP (TetOn-Rv1264)
TetR
+ Atc
RNAP
Promoter+ Tet Operator
Cyclase Domain
6X His
Catalytically Active Rv1264
ATP
cAMP
B.
Anti-His
Anti-GroEL2
1 2 3 4 5 6 7 8
1: Tet-On Rv1264 +EtOH
2: Tet-On Rv1264 +Atc
3: Tet-On Rv1264$_{D265A}$ +EtOH
4: Tet-On Rv1264$_{D265A}$ +Atc
5: Tet-On Rv1264 prpD'::GFP +EtOH
6: Tet-On Rv1264 prpD'::GFP +Atc
7. Tet-On Rv1264$_{D265A}$ prpD'::GFP +EtOH
8. Tet-On Rv1264$_{D265A}$ prpD'::GFP +Atc
C.
Total cAMP
24 hours
pmol cAMP/10^8 CFU
**
ns
EtOH
V-59 (10 μM)
Atc (500 ng/mL)
D.
TetOn-Rv1264$_{D265A}$
7H12+Cholesterol
OD $_{600}$
EtOH
V-59 (10 μM)
Atc (500 ng/mL)
Atc (50 ng/mL)
Day
E.
TetOn-Rv1264$_{D265A}$
[4-^{14}C]-Cholesterol
% $^{14}CO_2$ release
ns
EtOH
Atc (500 ng/mL)
F.
TetOn-Rv1264$_{D265A}$
prpD'::GFP
%GFP MFI
**
ns
ns
EtOH
V-59 (10 μM)
Atc (500 ng/mL)
Atc (50 ng/mL)

Fig. S4. Construction and validation of TetOn-cAMP constructs. (**A**) Schematic illustrating the domains of the native Rv1264 adenylyl cyclase (left) and the design of the TetOn-cAMP construct (right). The TetOn-cAMP construct contains the minimum-necessary cyclase domain of Rv1264, and lacks the pH-sensitive inhibitory domain of the native Rv1264 protein. Expression of the cyclase domain is under control of a TetOn promoter. Upon treatment with Atc, release of the tetracycline repressor (TetR) causes initiation of transcription of the Rv1264 catalytic domain. The C-terminal end of the cyclase domain is His-tagged to allow immunoblotting. (**B**) Immunoblots of bacterial lysates confirm that the TetOn constructs are expressed in the presence of Atc and not the vehicle control EtOH. The anti-His blot detects the Rv1264 cyclase domain and the anti-GroEL2 blot is the loading control. (**C**) Impact of inducing the TetOn-$Rv1264_{D265A}$ construct in media containing cholesterol and acetate. Cultures were treated with one dose of EtOH, V-59 (10 μM), or Atc at the indicated concentrations and samples were collected 24 hours later for ELISA. Data are displayed as total cAMP per 10^8 Mtb. Data are from two independent experiments with two technical replicates each, shown as means ± SD (**$P < 0.01$, One-way ANOVA with Dunnett's multiple comparisons test). (**D**) Effect of inducing TetOn-$Rv1264_{D265A}$ on the growth of Mtb in 7H12+cholesterol media, monitored by serial OD measurements. EtOH, V-59 (10 μM), or Atc at the indicated concentrations were added initially and every three days for the duration of the experiment. Data are from one experiment with three technical replicates, shown as means ± SD. (**E**) Catabolic release of $^{14}CO_2$ from [4-^{14}C]-cholesterol in media containing cholesterol and acetate. The TetOn-$Rv1264_{D265A}$ strain was treated with EtOH, V-59 (10 μM), or Atc at the indicated concentrations overnight and again one hour prior to the beginning of the experiments. Data are from two independent experiments with three technical replicates each, normalized to OD and quantified relative to EtOH vehicle control. Shown as means ± SD (not significant, Student's *t* test). (**F**) Relative GFP signal from the *prpD'*::GFP reporter in response to inducing TetOn-$Rv1264_{D265A}$ in media containing cholesterol and acetate. Cultures were treated with EtOH, V-59 (10 μM), or Atc at the indicated concentrations. Data are normalized to EtOH vehicle control (**$P < 0.01$, One-way ANOVA with Dunnett's multiple comparisons test). GFP MFI was quantified from 10,000 $mCherry^+$ Mtb. Data are from two independent experiments with two technical replicates each, shown as means ± SD.
