# Supplementary material for: Pharmacological and genetic activation of cAMP synthesis disrupts cholesterol utilization in Mycobacterium tuberculosis
Source: PLoS Pathog. 2022 Feb 8;18(2):e1009862. doi: 10.1371/journal.ppat.1009862 (PMC8856561; doi:10.1371/journal.ppat.1009862)
Supplement: S5 Fig — (A) Inhibitory activity of V-59 against WT or ΔMt-Pat in 7H12+cholesterol media. Data shown are from one experiment with two technical replicates. Symbols are mean data points, and curves display nonlinear fit of dose-response. (B) Relative GFP signal from the prpD’::GFP reporter in WT versus ΔMt-Pat strains carrying the TetOn-cAMP construct in media containing cholesterol and acetate. Cultures were treated in parallel with EtOH, V-59 (10 μΜ), or Atc at the indicated concentrations. Data are normalized to EtOH vehicle control in each strain. GFP MFI was quantified from 10,000 mCherry+ Mtb. Data are from one experiment with two technical replicates, shown as means ± SD. (C) Effect of inducing TetOn-cAMP on the growth of Mtb in 7H12+cholesterol+acetate media. EtOH, V-59 (10 μM), or Atc at the indicated concentrations were added initially and every three days for the duration of the experiment. Data are from one experiment with three technical replicates, shown as means ± SD. (D) Relative GFP signal from the prpD’::GFP reporter in Mtb treated with V-59 or DMSO, in 7H12 media supplemented with C17:1 or propionate. Data shown normalized to WT+DMSO. GFP MFI was quantified from 10,000 mCherry positive Mtb. Data are from one experiment with two technical replicates, shown as means ± SD. (E) Effect of mCLB073 treatment combined with a sub-optimal dose of rifampicin (RIF) in BALB/c mice infected by the aerosol route. Data are from one experiment with 10 mice per group (**P < 0.01, Mann-Whitney test). Data are shown as means ± SEM. (F) Quantification of cAMP in human cell lines treated with V-59 (10 μM) or DMSO control. Data are from one experiment with two technical replicates, and are shown as means ± SD. (PDF) [file ppat.1009862.s005.pdf]

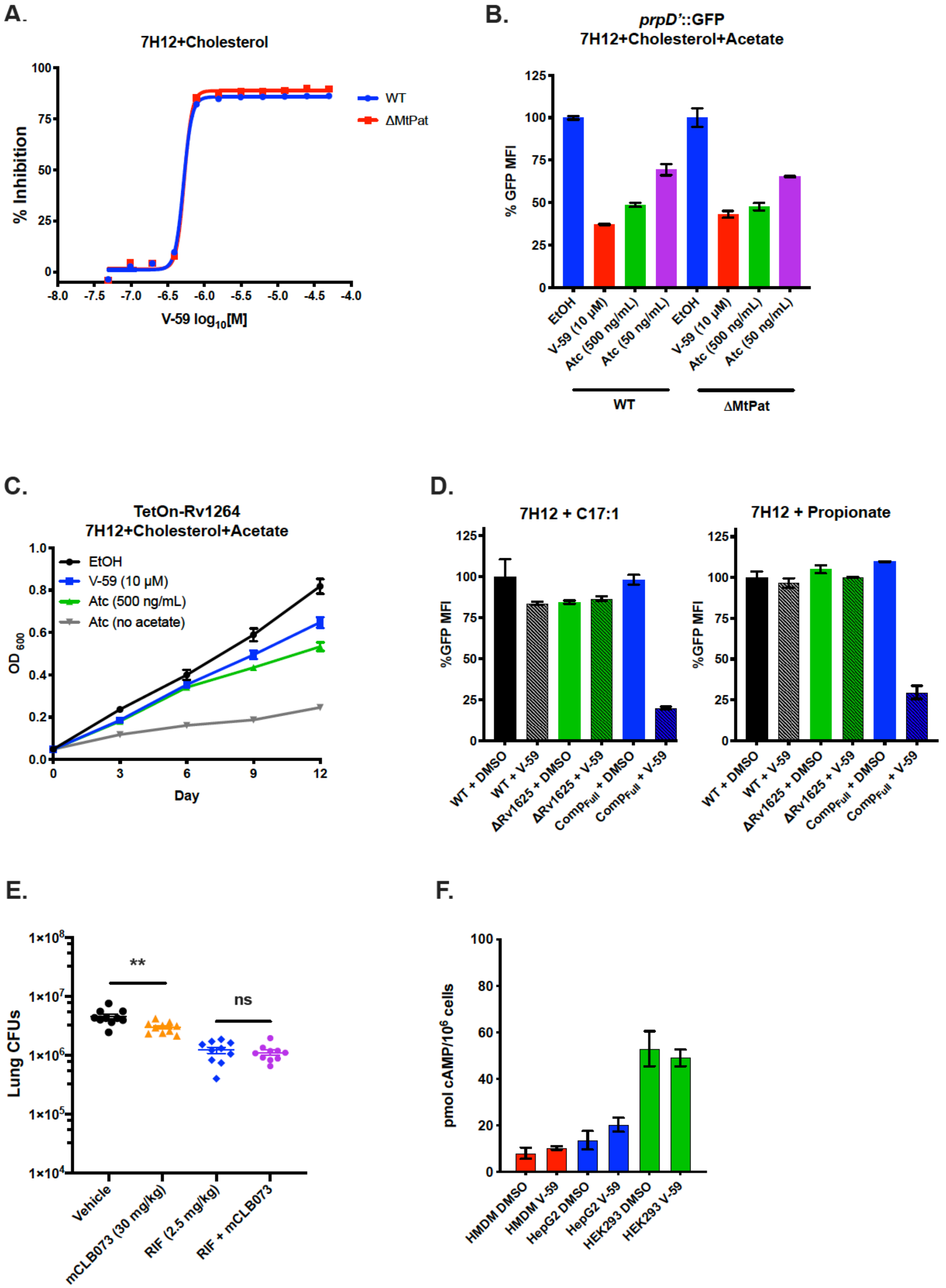

A.
7H12+Cholesterol
% Inhibition
V-59 log10[M]
WT
ΔMtPat
B.
prpD'::GFP
7H12+Cholesterol+Acetate
% GFP MFI
EtOH
V-59 (10 μM)
Atc (500 ng/mL)
Atc (50 ng/mL)
WT
ΔMtPat
C.
TetOn-Rv1264
7H12+Cholesterol+Acetate
OD 600
Day
EtOH
V-59 (10 μM)
Atc (500 ng/mL)
Atc (no acetate)
D.
7H12 + C17:1
7H12 + Propionate
%GFP MFI
WT + DMSO
WT + V-59
ΔRv1625 + DMSO
ΔRv1625 + V-59
CompFull + DMSO
CompFull + V-59
E.
Lung CFUs
**
ns
Vehicle
mCLB073 (30 mg/kg)
RIF (2.5 mg/kg)
RIF + mCLB073
F.
pmol cAMP/10^6 cells
HMDM DMSO
HMDM V-59
HepG2 DMSO
HepG2 V-59
HEK293 DMSO
HEK293 V-59

Fig. S5. Activating cAMP synthesis inhibits lipid metabolism in an Mt-Pat independent mechanism, and can inhibit fatty acid utilization without increasing antibiotic tolerance or increasing mammalian cAMP synthesis. (**A**) Inhibitory activity of V-59 against WT or ΔMt-Pat in 7H12+cholesterol media. Data shown are from one experiment with two technical replicates. Symbols are mean data points, and curves display nonlinear fit of dose-response. (**B**) Relative GFP signal from the *prpD'*::GFP reporter in WT versus ΔMt-Pat strains carrying the TetOn-cAMP construct in media containing cholesterol and acetate. Cultures were treated in parallel with EtOH, V-59 (10 μM), or Atc at the indicated concentrations. Data are normalized to EtOH vehicle control in each strain. GFP MFI was quantified from 10,000 $mCherry^{+}$ Mtb. Data are from one experiment with two technical replicates, shown as means ± SD. (**C**) Effect of inducing TetOn-cAMP on the growth of Mtb in 7H12+cholesterol+acetate media. EtOH, V-59 (10 μM), or Atc at the indicated concentrations were added initially and every three days for the duration of the experiment. Data are from one experiment with three technical replicates, shown as means ± SD. (**D**) Relative GFP signal from the *prpD'*::GFP reporter in Mtb treated with V-59 or DMSO, in 7H12 media supplemented with C17:1 or propionate. Data shown normalized to WT+DMSO. GFP MFI was quantified from 10,000 mCherry positive Mtb. Data are from one experiment with two technical replicates, shown as means ± SD. (**E**) Effect of mCLB073 treatment combined with a sub-optimal dose of rifampicin (RIF) in BALB/c mice infected by the aerosol route. Data are from one experiment with 10 mice per group (**$P < 0.01$, Mann-Whitney test). Data are shown as means ± SEM. **(F)** Quantification of cAMP in human cell lines treated with V-59 (10 μM) or DMSO control. Data are from one experiment with two technical replicates, and are shown as means ± SD.
