# Supplementary material for: Pharmacological and genetic activation of cAMP synthesis disrupts cholesterol utilization in Mycobacterium tuberculosis
Source: PLoS Pathog. 2022 Feb 8;18(2):e1009862. doi: 10.1371/journal.ppat.1009862 (PMC8856561; doi:10.1371/journal.ppat.1009862)
Supplement: S1 Table — Chemical structure of previously published single-step cholesterol breakdown inhibitors, and resynthesized analogs of previously described Rv1625c-dependent inhibitors. MW, molecular weight; —, not determined; EC50, half-maximal effective concentration; hERG, human ether-à-go-go-related gene; CC50, 50% cytotoxic concentration; PPB, plasma protein binding; IC50, half-maximal inhibitory concentration; Cyp, cytochrome P450; ER, extraction ratio; PSA, polar surface area. (PDF) [file ppat.1009862.s006.pdf]

	Rv1625c-Independent Cholesterol Breakdown Inhibitors		Analogs of Previously Published Rv1625c-Dependent Inhibitors	
	V-13-012725	V-13-011503	sCEB942	mCCY224
	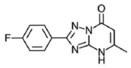	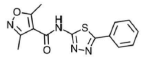	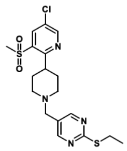	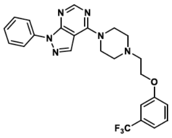
MW	244	276	426	468
Intramacrophage EC_{50} (μM)	25	25	4.16	2.96
Cholesterol EC_{50} (μM)	1.4	5	0.67	0.35
7H9 OADC EC_{50} (μM)	>50	>50	>20	9.56
hERG (%)	--	--	--	89.09
HepG2 CC_{50} (μM)	--	--	--	20
Cyp IC50 (μM, 2C19/2C9)	--	--	--	9.55/23.29
Mouse PPB (%)	--	--	89.61	99.96
Caseum binding (% unbound)	--	--	3.34	<<0.01
ER (mouse/human)	--	--	0.79/0.56	0.48/0.43
Solubility (mM, pH 6.8)	--	--	183.09	1
Time over EC_{50}	--	--	--	>12h
CLogP	--	--	3.42	4.04
PSA	--	--	109.7	59.3

Table S1. V-59 is structurally distinct from other cholesterol utilization inhibitors. Chemical structure of previously published single-step cholesterol breakdown inhibitors, and resynthesized analogs of previously described Rv1625c-dependent inhibitors. MW, molecular weight; --, not determined; EC_{50}, half-maximal effective concentration; hERG, human ether-à-go-go-related gene; CC_{50}, 50% cytotoxic concentration; PPB, plasma protein binding; IC_{50}, half-maximal inhibitory concentration; Cyp, cytochrome P450; ER, extraction ratio; PSA, polar surface area.
