# Supplementary material for: Pharmacological and genetic activation of cAMP synthesis disrupts cholesterol utilization in Mycobacterium tuberculosis
Source: PLoS Pathog. 2022 Feb 8;18(2):e1009862. doi: 10.1371/journal.ppat.1009862 (PMC8856561; doi:10.1371/journal.ppat.1009862)
Supplement: S2 Table — MW, molecular weight; —, not determined; EC50, half-maximal effective concentration; IC50, half-maximal inhibitory concentration; ADME, absorption, distribution, metabolism, excretion; PPB, plasma protein binding; ER, extraction ratio; PO, per os. (PDF) [file ppat.1009862.s007.pdf]

	mCLF024	mCLF025
	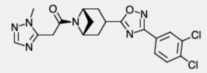	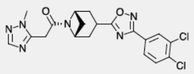
**MW**	**433.29**	**422.29**
	***In vitro* potency assays**	
**Macrophages EC_{50} (μM)**	**97**	**>30,000**
**Cholesterol EC_{50} (nM)**	**49**	**>30,000**
	**Toxicity**	
**hERG IC_{50} (μM)**	**11.8**	**--**
	**ADME**	
**PBB mouse (% bound)**	**97.8**	**--**
**Caseum binding (% unbound)**	**<0.01**	**--**
**ER mouse/human**	**<0.3/<0.3**	**--**
**Solubility (μM, pH 6.8)**	**94.0**	**--**

Table S2. Structures and activities of isomer compounds. MW, molecular weight; --, not determined; EC_{50}, half-maximal effective concentration; IC_{50}, half-maximal inhibitory concentration; ADME, absorption, distribution, metabolism, excretion; PPB, plasma protein binding; ER, extraction ratio; PO, per os.
