# Supplementary material for: Pharmacological and genetic activation of cAMP synthesis disrupts cholesterol utilization in Mycobacterium tuberculosis
Source: PLoS Pathog. 2022 Feb 8;18(2):e1009862. doi: 10.1371/journal.ppat.1009862 (PMC8856561; doi:10.1371/journal.ppat.1009862)
Supplement: S3 Table — (PDF) [file ppat.1009862.s008.pdf]

Strain	Location of antibiotic resistance cassette insertion	Background	Antibiotic Resistance
Tn::Rv1625c	Mycomar transposon insertion at 904bp of *Rv1625c*	M. tuberculosis CDC1551	Kanamycin
ΔRv1625c	(92-1256bp) of *Rv1625c*	M. tuberculosis CDC1551	Hygromycin
ΔMt-Pat	(21-981bp) of *Rv0998*	M. tuberculosis CDC1551	Hygromycin

Strain	Plasmid Constructs	Background	Antibiotic Resistance
2xRv1625*	pMV306+aacC4 (apramycin resistance)+ hsp60'::Rv1625c (1-1329bp)	WT M. tuberculosis CDC1551	Apramycin
Comp$_{Full}$*	pMV306 + aacC4 (apramycin resistance) + hsp60'::Rv1625c (1-1329bp)	ΔRv1625c	Hygromycin, Apramycin
Comp$_{D204}$*	pMV306+aacC4 (apramycin resistance) + hsp60'::Rv1625c (1-612bp)	ΔRv1625c	Hygromycin, Apramycin
TetOn-cAMP**	pDE1264 (pDO23A+Rv1264 (627-1191bp)) + pEN41A-T10M + pEN12A-p606 + pDE43-MEH	WT M. tuberculosis CDC1551	Hygromycin
TetOn-Rv1264$_{D265A}$**	pDE1264$_{D265A}$ (pDO23A+Rv1264$_{D265A}$(624-1191bp))+ pEN41A-T10M + pEN12A-p606 + pDE43-MEH	WT M. tuberculosis CDC1551	Hygromycin
prpD'::GFP	pCherry3 + kanamycin resistance + prpD'::GFP + smyc'::mCherry	WT M. tuberculosis CDC1551	Kanamycin
ΔRv1625c prpD'::GFP	pCherry3 + kanamycin resistance + prpD'::GFP + smyc'::mCherry	ΔRv1625c	Kanamycin
Comp$_{Full}$ prpD'::GFP	pCherry3 + zeocin resistance + prpD'::GFP + smyc'::mCherry	Comp$_{Full}$	Hygromycin, Apramycin, Zeocin
Comp$_{D204}$ prpD'::GFP	pCherry3 + zeocin resistance + prpD'::GFP + smyc'::mCherry	Comp$_{D265A}$	Hygromycin, Apramycin, Zeocin
TetOn-cAMP prpD'::GFP	TetOn-cAMP + prpD'::GFP + smyc'::mCherry	WT M. tuberculosis CDC1551	Hygromycin
ΔMt-Pat TetOn-cAMP prpD'::GFP	TetOn-cAMP + kanamycin resistance + prpD'::GFP + smyc'::mCherry	ΔMt-Pat	Hygromycin, Kanamycin
TetOn-1264$_{D265A}$ prpD'::GFP	TetOn-Rv1264$_{D265A}$ + prpD'::GFP + smyc'::mCherry	WT M. tuberculosis CDC1551	Hygromycin

*Apramycin gene sourced from Consaul SA, Pavelka MS Jr. Use of a novel allele of the Escherichia coli aacC4 aminoglycoside resistance gene as a genetic marker in mycobacteria. FEMS Microbiol Lett. 2004 May 15;234(2):297-301. doi: 10.1016/j.femsle.2004.03.041. PMID: 15135536.
**Plasmids for Gateway cloning to construct TetOn-cAMP and TetOn-Rv1264$_{D265A}$ developed from Klotzsche M, Ehrt S, Schnappinger D. Improved tetracycline repressors for gene silencing in mycobacteria. Nucleic Acids Res. 2009 Apr;37(6):1778-88. doi: 10.1093/nar/gkp015. PMID: 19174563.

Table S3. Mtb strains used in these experiments.
