# Supplementary material for: Pharmacological and genetic activation of cAMP synthesis disrupts cholesterol utilization in Mycobacterium tuberculosis
Source: PLoS Pathog. 2022 Feb 8;18(2):e1009862. doi: 10.1371/journal.ppat.1009862 (PMC8856561; doi:10.1371/journal.ppat.1009862)
Supplement: S1 File — (PDF) [file ppat.1009862.s009.pdf]

Compound optimization

To identify mCLB073, the potency, safety, and pharmacokinetic properties of the oxadiazole series represented by V-59 was optimized. The cellular activity of V-59 analogs were assessed based on *in vitro* potency against Mtb in cholesterol-based media and intramacrophage assays, and cytotoxicity counterscreens against mammalian cells to ensure high selectivity.

Compound formulations for *in vivo* experiments

V-59 and isoniazid (Sigma Aldrich) were solubilized in 10% DMSO, 70% PEG 300, and 20% D5W (Dextrose 5% in ddH_2O) with heating and sonication for intranasal BALB/c and C3HeB/FeJ experiments. For mCLB073 studies, compounds were solubilized at the indicated concentrations in 0.5% methyl cellulose, 0.5% Tween-80 (intranasal infection experiment) or 10% 2-hydroxypropyl-β-cyclodextrin + 10% lecithin (aerosol infection experiment) to obtain a fine suspension. Doses of 0.1mL were delivered by oral gavage.

In vitro cytotoxicity assay

In vitro cytotoxicity was evaluated in HepG2 cells (ATCC HB-8065, human liver) and in HEK293 cells (ATCC CRL-3216, human kidney). Cells were cultured in Dulbecco's modified Eagle's medium (DMEM) supplemented with 10% fetal bovine serum (FBS) and maintained in a humidified incubator (37°C in 5% CO_2). Cells were harvested by trypsin treatment, collected by centrifugation, resuspended in assay medium with FBS (2 %) at the cell density of $7.5x10^4$ (HEK293T) or $5.0x10^4$ (HepG). Cell suspensions were dispensed (5 µL per well) into 1,536-well

white microtiter plates pre-spotted with test compounds (Echo Labcyte), covered with metal lids (GNF), and incubated for 72 hours at 37°C. After 72 hours, CellTiterGlo (Promega) reagent was diluted 1:1 with ultra-pure water and dispensed into the plates (2 uL per well). Luminescence was measured with a microplate reader (EnVision), and the cytotoxic concentration, half maximal effect (CC_{50}) was calculated for each compound with Genedata software. Test compounds were normalized to DMSO only wells, with puromycin (10 µM) as a positive control.

Manual patch clamp assay for hERG (human ether-a-go-go-related gene) potassium channel inhibition

hERG inhibition was evaluated in CHO cells that stably express the hERG potassium channel (Sophion Biosciences). Cells were cultured in F12 Hams medium supplemented with 10% FBS, Hygromycin (100 µg/ml) (Thermo Fisher Scientific), and G418 (100 µg/ml) (Thermo Fisher Scientific) and maintained in a humidified incubator (37°C in 5% CO_2). The test compounds were dissolved in DMSO (100 %) to obtain sub-stock solutions for different test concentrations and further diluted into media to achieve final concentrations for testing in cell culture. Final DMSO concentration did not exceed 0.30% in the compound treated, vehicle control, or positive (Amitriptyline) control.

The patch clamp assay was conducted with the extracellular solution (mM)： NaCl 145, KCl 4, $CaCl_2$ 2, $MgCl_2$ 1, glucose 10, HEPES 10, pH = 7.4 and Osmolarity 290~320 mOsm, and the intracellular solution (mM)： KOH 31.25, KCl 120, EGTA 10, $MgCl_2$ 1.75, $CaCl_2$ 5.374, HEPES 10, Na-ATP 4, pH=7.2 and Osmolarity 280~310 mOsm. Compounds were tested at room

temperature using a USB amplifier (HEKA Elektronik). Output signals were low-pass filtered at 3 KHz and controlled with PatchMaster software (HEKA Elektronik). For quality control, the minimum seal resistance was set at 500 MOhms, and minimum specific hERG current (pre-compound) was 0.4 nA. Micropipettes (Harvard Apparatus) were pulled with a programmable micropipette puller (NARISHIGEPC-10Puller). The pipette tip resistance was between 2 ~MOhms The recorded cells were continuously perfused with extracellular solution (Octaflow) at a flow rate of ~1 ml/min mounted on the stage of an upright microscope (Nikon). The perfusion tip was manually positioned and five concentrations were tested in duplicate. Voltage command protocol: From the holding potential of -80 mV, the voltage was first stepped to 60 mV for 0.850 sec to open the hERG channels. Then the voltage was stepped down to -50 mV for 1.275 sec, generating a tail current, which was measured and collected for data analysis. Finally, the voltage was stepped back to the holding potential (-80 mV) and repeated every 15,000 msec. This command protocol was performed continuously during the test (vehicle control, test compounds). Compound application: During the initial recording period, the peak current amplitude was monitored until stable (< 5% change) for 5~10 sweeps. Once stabilized, drug perfusion started with the lowest concentration and continued until the peak current stabilized across 5 sweeps, or 5 minutes if peak current did not change.

Data analysis was carried out using PatchMaster software (HEKA Elektronik) Excel 2013 (Microsoft) and Prism 5 (GraphPad). Normalized current values for each test compound concentration were calculated from recorded current responses: (peak current measured under compound perfusion /peak current measured with vehicle perfusion) ×100%. The percent inhibition values for each test article concentration were calculated from recorded current

responses according to the formula: (1 - peak current measured under compound perfusion / peak current measured with vehicle perfusion)×100%. The final IC_{50} was determined with curve fitting. I/I control =Bottom + (Top-Bottom)/(1+10^((LogIC50-X)*Hill Slope)) where X is the logarithm of concentration, I/I control is the normalized peak current amplitude, Top is 1 and Bottom is equal to 0.

Plasma protein binding assay

Plasma protein binding to compounds was quantified using human plasma (BioIVT) or plasma from CD-1 mice (Beijing Vital River Laboratory Animal Technology Co., Ltd) using a rapid equilibrium diffusion (RED) method. Equilibrium of free compound is achieved by the diffusion of the unbound compound across the (10-14 kDa MWCO) dialysis membrane in 96-well Micro-Equilibrium Dialysis Devices (HTDialysis). Briefly, sterile plasma containing test compounds was added to the matrix side of chamber in a commercial dialysis plate with reconstituted dialysis strips. Isotonic sodium phosphate buffer was added to the peripheral chamber of the dialysis plate and the unit was placed in a humidified incubator at 37 °C with 5% CO_2 on a shaking platform at 100 rpm for 4 hours. Following dialysis, 50 µL aliquots of the samples were taken from the buffer side and the matrix side of the dialysis device and quenched with acetonitrile (100%) containing internal standards tolbutamide (200 ng/mL), labetalol (200 ng/mL) and metformin (50 ng/mL). The concentration of free and bound test compound was determined by LC/MS analysis. The % Unbound, % Bound, and % Recovery values were calculated using the following equations: %Unbound=100 × F / T and %Bound = 100 - %Unbound where [F] is the analyte concentration or peak area ratio of analyte/internal standard in the buffer on the peripheral side of the membrane, [T] is the analyte concentration or peak area ratio of analyte/internal standard on the matrix side

of the membrane, and [T0] is the analyte concentration or the peak area ratio of analyte/internal standard in the loading matrix sample at time zero.

Caseum binding assay

Caseum binding was quantified using caseum harvested from rabbit Mtb granuloma lesions as described (*1*). Briefly, sterile caseum was spiked with test compounds and added to the matrix chamber of a commercial plate based micro-equilibrium dialysis device (Thermo Scientific Pierce RED Device). Isotonic sodium phosphate buffer was added to the peripheral chamber of the RED device and the plate was incubated at 37°C for 4 hours. Equilibrium of free compound is achieved by the diffusion of the unbound compound across the (8 kDa MWCO) dialysis membrane. Following incubation, aliquots of the isotonic buffer and the plasma were taken at pre-determined time points and the concentration of free and bound test compound was determined by LC/MS analysis.

CYP Inhibition evaluation in human liver microsomes

Inhibition of five separate P450 monooxygenases was quantified using human liver microsomes (Corning). Briefly, microsomes were prepared in potassium phosphate buffer (100 mM) pH 7.2 containing phenacetin (10 μM), diclofenac (5 μM), S-mephenytoin (30 μM), dextromethorphan (5 μM), and midazolam (2 μM) which are substrates for the 1A2, 2C9, 2C19, 2D6, and 3A4 monooxygenase enzymes, respectively. Human liver microsomes were loaded into wells of an incubation plate and prewarmed to 37°C. NADPH was added to the final concentration of 10 mmol to all wells and the plate was incubated for 10 minutes at 37°C. The reactions were terminated by

adding acetonitrile (100%), containing the internal standards tolbutamide (200 ng/mL) and labetalol (200 ng/mL). Protein was removed from the samples by centrifugation and the P450 monooxygenase enzymatic products were quantified by LC/MS/MS analysis. The concentration of the enzymatic products in the reaction mixture was used to calculate the percent of inhibition vehicle control versus the test compound concentrations (0, 0.05, 0.15, 0.5, 1.5, 5.0, 15.0 and 50 μM). Data was fit using a non-linear regression analysis and the IC_{50} values were determined using 3- or 4- parameter logistic equation. IC_{50} values were reported as ">50 μM" when % inhibition at the highest concentration (50 μM) is less than 50%.

Equation for three parameters logistic sigmoidal curve:

$$y = \frac{max}{1+\left(\frac{x}{IC_{50}}\right)^{-hillslope}}$$

Equation for four parameters logistic sigmoidal curve:

$$y = min + \frac{max-min}{1+\left(\frac{x}{IC_{50}}\right)^{-hill\ slope}}$$

Liver microsomal stability

Liver microsomes from mouse (Xenotech), human (Corning) rat (Xenotech) and dog (Xenotech) were prepared in potassium phosphate buffer (100 mM) pH 7.2. Experimental compounds were dissolved in DMSO (100%) to a stock concentration of 10 mmol. A working compound solution was made by diluting 5 μL of compound stock into 495 μL of acetonitrile (100%) to the final concentration of compound (100 μM) and acetonitrile (99%). Similarly, positive control compounds (Testosterone, Dichlofenac, and Propafenone) were dissolved in DMSO (100% to a

stock concentration of 10 mM in DMSO, and were diluted with 495 µL of acetonitrile (100%) to the final concentration of compound (100 µM) and acetonitrile (99%). NADPH•4Na was dissolved into a 10 mM $MgCl_2$ solution (10 unit/mL) generating as a 10x cofactor stock. Pre-warmed incubation plates were filled with 445 µL potassium phosphate buffer and incubated for 10 min at 37°C with constant shaking. Liver microsomes (54 µL) liver and NADPH stock (6 µL) were added to the plates. Working solution of compounds (5 µL) was added into the incubation plates containing microsomes and mixed thoroughly before incubating at 37°C for 60 min under constant shaking at 100 rpm. Reactions were stopped by the addition of 100 µl cold acetonitrile (100%) containing the internal standards tolbutamide (200 ng/mL) and labetalol (200 ng/mL). All plates were shaken for 10 min, then centrifuged at 4000 rpm for 20 minutes at 4°C. The cell free supernatant was transferred into HPLC water and mixed by shaking the plate for 10 min minutes prior to metabolite quantification by LC-MS/MS analysis.

The equation of first order kinetics was used to calculate $T_{1/2}$ and $CL_{int(mic)}$ (µL/min/mg):

Equation of first order kinetics:

$$C_t = C_0 \cdot e^{-k_e \cdot t}$$

$$when\ C_t = \frac{1}{2} C_0 \ ,$$

$$T_{\frac{1}{2}} = \frac{Ln2}{k_e} = \frac{0.693}{k_e}$$

$$CL_{int(mic)} = \frac{0.693}{In\ vitro\ T_{\frac{1}{2}}} \cdot \frac{1}{mg/mL\ microsomal\ protein\ in\ reaction}$$

$$CL_{int(liver)} = CL_{int(mic)} \cdot \frac{mg\ microsomes}{g\ liver} \cdot \frac{g\ liver}{kg\ body\ weight}$$

$T_{1/2}$ is half life and $CL_{int\,(\,mic)}$ is the intrinsic clearance

$CL_{int\,(\,mic)}$ = 0.693/half life/mg microsome protein per mL

$CL_{int(liver)} = CL_{int\,(\,mic)}$ * mg microsomal protein/g liver weight * g liver weight/kg body weight

Extraction ratio calculation

Extraction ratio (ER) = CLH/QH= CLint(liver) / (CLint(liver) + Qh)

Qh is Hepatic Blood Flow (mL/min/kg)

Solubility

Stock solutions containing compound of interest (10 mM) were prepared in DMSO (100%) and diluted to a final concentration (0.2 mM) in phosphate buffer solution (50 mM) pH 6.8. The sample was loaded in Mini-UniPrep vials containing 0.2 µm filters (GE Halthcare), vortexed for 2 mins and shaken for 24 hours at room temperature. The Mini-UniPrep was compressed and the resulting filtrates were analyzed by HPLC/UPLC system to calculate the concentration with standard curve (1, 20, and 200 µM).

Time over EC_{50} determination

The pharmacokinetic profile of mCLB073 was determined in male CD-1 mice after single dose oral administration (20 mg/kg). A 4 mg/ml stock solution of mCLB073 was prepared in 75% PEG 300, 25% D5W with vortexing to dissolve into solution. An aliquot of the dose solutions was taken

before the dosing and after dosing, and stored at approximately –20°C for subsequent analysis. Blood samples were collected at standard time points (0.25h, 1h, 3h, 5h, 8h, 24h) through a capillary via a retro orbital bleed. At each time point, approximately 100μL of blood samples were collected from 3 mice per group. The blood was heparin treated and within 1 hour of collection the blood samples were centrifuged at 2500x g for 15 minutes at 4°C to collect the plasma for quantification of drug concentration via LC/MS. Time over EC_{50} was calculated by multiple EC_{50} with molecular weight. The equation is: EC_{50} line (ng/ml) = molecular weight (g/mol) × EC_{50} (μM, μmol/L).

Western blot analysis

To confirm expression of His-tagged Rv1264 and $Rv1264_{D265A}$ in the Tet-On Mtb strains, bacteria were grown to an OD of 0.6 in 7H9OADC and then inoculated into 7H12+acetate containing EtOH or Atc (500 ng/mL) at an OD of 0.15. The following day, an equivalent number of bacteria were pelleted for each strain and treatment, and the pellets were fixed in paraformaldehyde (4%) for one hour and stored at -80°C. Fixed Mtb pellets were suspended in SDS (1%) and probe sonicated (3 x 1 minute cycles). Bacterial debris was removed by centrifugation, and the supernatants were suspended in SDS PAGE loading buffer and boiled for 30 minutes with periodic vortexing. Equivalent volumes of each sample were resolved by SDS-PAGE gel and transferred to nitrocellulose membranes. Western blotting was performed using either mouse anti-5His (Qiagen), or mouse anti-Mycobacterium tuberculosis GroEL2 (BEI Resources, Clone IT-70) primary antibody, and HRP-conjugated goat anti-mouse IgG (Jackson ImmunoResearch) secondary

antibody. Chemiluminescent substrate (Thermo Fisher Scientific) was added and Westerns were imaged by film for anti-His or by ChemiDoc (Bio-Rad) for anti-GroEL2.

cAMP production by cultured human cells.

cAMP production was evaluated in HepG2 cells (ATCC HB-8065, human liver), HEK293 cells (ATCC CRL-3216, human kidney) and human monocyte derived macrophages (HMDM). HepG2 and HEK293 cells were cultured in DMEM supplemented with 10% FBS and maintained in a humidified incubator (37°C in 5% CO2). HMDM cells derived from human peripheral blood mononuclear cells (PBMCs) that were obtained from Elutriation Core Facility, University of Nebraska Medical Center. HMDM cells were cultured in DMEM supplemented with 10% human serum, L-glutamine (2 mmol), sodium pyruvate (1 mmol), penicillin (100 U/mL), and streptomycin (100 µg/mL) (Corning) and maintained in a humidified incubator (37°C in 5% CO_2). Cells were cultured at a density of 1e6 per T-25 flask and were treated with V-59 (10 µM) for 24 hours before harvesting the cells with trypsin treatment (HepG2 and HEK293) or scraping into cold PBS (HMDM). Cells were harvested by centrifugation and cAMP levels were quantified from the cell pellet following, resuspension in lysis buffer (0.1M HCl, 1% Triton X-100 in ddH_2O). The cell-free lysates were used to measure internal cAMP by ELISA (Enzo Life Sciences).

References

1. J. P. Sarathy, F. Zuccotto, H. Hsinpin, L. Sandberg, L. E. Via, G. A. Marriner, T. Masquelin, P. Wyatt, P. Ray, V. Dartois, Prediction of Drug Penetration in Tuberculosis Lesions. *ACS Infectious Diseases* **2**, 552-563 (2016).
