# Supplementary material for: Pharmacological and genetic activation of cAMP synthesis disrupts cholesterol utilization in Mycobacterium tuberculosis
Source: PLoS Pathog. 2022 Feb 8;18(2):e1009862. doi: 10.1371/journal.ppat.1009862 (PMC8856561; doi:10.1371/journal.ppat.1009862)
Supplement: S2 File — (PDF) [file ppat.1009862.s010.pdf]

General chemistry information

All solvents and reagents were purchased (Sigma Aldrich or Thermo Fisher) and used without further purification. Analytical thin-layer chromatography (TLC) was conducted on Silica gel 60 F254 plates (EMD Chemicals), and compounds were visualized with UVG-11 mineralight UV lamp (UVP Inc) at 254 nm. Flash column chromatography was performed with silica gel (Combi Flash). ^{1}H, ^{13}C and ^{19}F Nuclear Magnetic Resonance (NMR) spectra were obtained on a Bruker400 or Bruker500 instruments recorded in $CDCl_3$ or d6-DMSO. Signal splitting patterns were described as singlet (s), doublet (d), doublet of doublet (dd), doublet of triplet (dt), triplet (t), or multiplet (m), with coupling constants (J) in hertz. High resolution mass spectra were performed on an Electron Spray Injection (ESI) mass spectrometer. Liquid chromatography mass spectrometry (LC/MS) was performed on Agilent 1100 series equipped with eclipse plus C18 analytical column (5 µm, 4.6×50 mmol). The elution condition was a linear gradient increase of solvent B from 10% to 90% over 6 minutes at a flow rate of 1 mL/min.

Compound optimization

To identify mCLB073, the potency, safety, and pharmacokinetic properties of the oxadiazole series represented by V-59 was optimized. The cellular activity of V-59 analogs were assessed based on *in vitro* potency against Mtb in cholesterol-based media and intramacrophage assays, and cytotoxicity counter screens against mammalian cells to ensure high selectivity.

Compound formulations for *in vivo* experiments

V-59 and isoniazid (Sigma Aldrich) were solubilized in 10% DMSO, 70% PEG 300, and 20% D5W (Dextrose 5% in ddH_2O) with heating and sonication for intranasal BALB/c and C3HeB/FeJ

experiments. For mCLB073 studies, all compounds were solubilized at the indicated concentrations in 0.5% methyl cellulose, 0.5% Tween-80 to obtain a fine suspension. Doses of 0.1mL were delivered by oral gavage.

Compound synthesis and structural confirmation

1-(4-(3-(4-fluorophenyl)-1,2,4-oxadiazol-5-yl)piperidin-1-yl)-2-(4-methyl-1,2,5-oxadiazol-3-yl)ethan-1-one (V-59):

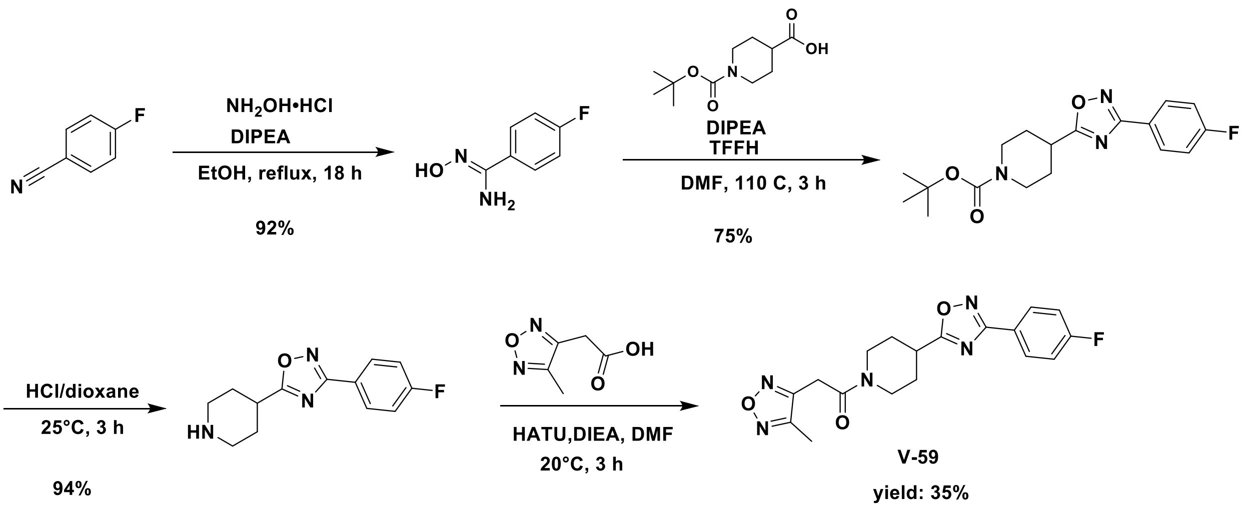

4-Fluoro-N'-hydroxy-benzamidine

To a solution of 4-Fluorobenzonitrile (4.8 g, 40 mmol, 1.0 *eq*) in EtOH (20 mL) was added NH_2OH•HCl (2.6 g, 40 mmol, 1.0 *eq*) and DIPEA (8.3 g, 64 mmol, 1.6 *eq*) at 0°C. The mixture was stirred and refluxed for 18 hours. LC/MS showed the starting material was consumed completely and the desired compound was detected. The reaction mixture was concentrated, and dissolved in ethyl acetate (80 mL), and extracted with water (2 × 100 mL). Then the organic layer was washed with brine (80 mL) and dried over Na_2SO_4. Then the mixture was filtered, and the filtrate was concentrated to afford 4-fluoro-N'-hydroxy-benzamidine (5.7 g, 36.9 mmol, 92% yield) as a white solid. LC/MS [ESI, M+1]: m/z 155.1.

***Tert*-butyl 4-(3-(4-fluorophenyl)-1,2,4-oxadiazol-5-yl)piperidine-1-carboxylate**

To a solution of 1-(*tert*-butoxycarbonyl)piperidine-4-carboxylic acid in DMF (30 mL) was added DIPEA(2.6 g, 20 mmol, 1.0 *eq*) and TFFH (5.3 g, 20 mmol, 1.0 *eq*). The mixture was stirred at 25 °C for 30 min, then 4-fluoro-N'-hydroxy-benzamidine (3.1 g, 20 mmol, 1.0 *eq*) was added. The mixture was stirred at 110 °C for 3 hours. LC/MS showed the starting material was completely consumed and the desired compound was detected. The reaction mixture was concentrated, and then dissolved in ethyl acetate (50 mL). The organic phase was washed with 1 N aqueous HCl solution (2 × 50 mL) and then the organic phase was dried over Na_2SO_4, and the filtrate was concentrated to afford *tert*-butyl 4-(3-(4-fluorophenyl)-1,2,4-oxadiazol-5-yl)piperidine-1-carboxylate (5.2 g, 15 mmol, 75% yield) as a white solid. LC/MS [ESI, M-55]: m/z 293.1.

3-(4-Fluorophenyl)-5-(piperidin-4-yl)-1,2,4-oxadiazole

HCl/dioxane (4.0 M, 20 mL, 5.3 *eq*) was added to 4-(3-(4-fluorophenyl)-1,2,4-oxadiazol-5-yl)piperidine-1-carboxylate (5.2 g, 15 mmol, 1.0 *eq*). The mixture was stirred at 25 °C for 3 hours. LC/MS showed the starting material was consumed completely and the desired compound was detected. The mixture was concentrated under vacuum to give 3-(4-fluorophenyl)-5-(piperidin-4-yl)-1,2,4-oxadiazole (HCl salt, 3.5 g, 14.1 mmol, 94% yield) as light brown solid. LC/MS [ESI, M+1]: m/z 248.2.

1-(4-(3-(4-fluorophenyl)-1,2,4-oxadiazol-5-yl)piperidin-1-yl)-2-(4-methyl-1,2,5-oxadiazol-3-yl)ethan-1-one (V-59):

To a solution of 3-(4-fluorophenyl)-5-(piperidin-4-yl)-1,2,4-oxadiazole (HCl salt, 1.4 g, 10 mmol, 1.0 *eq*) in DMF (20 mL) was added HATU (5.7 g, 15 mmol, 1.5 *eq*), 2-(4-methyl-1,2,5-oxadiazol-3-yl)acetic acid (1.7 g, 12 mmol, 1.2 *eq*) and DIPEA (3.9 g, 30 mmol, 5.2 mL, 3.0 *eq*). The mixture was stirred at 20 °C for 18 hours. The mixture was concentrated under vacuum and diluted with ethyl acetate (50 mL) and washed with water (2 × 100 mL). Then the organic layer was washed with brine (80 mL), dried over Na_2SO_4, and the mixture was filtered, and the filtrate was concentrated. The residue was purified by silica gel chromatography eluted with 0 - 50% ethyl acetate in hexane to afford 1-(4-(3-(4-fluorophenyl)-1,2,4-oxadiazol-5-yl)piperidin-1-yl)-2-(4-methyl-1,2,5-oxadiazol-3-yl)ethan-1-one (1.3 g, 3.5 mmol, 35% yield) as white solid. LC/MS [ESI, M+1]: m/z 372.2. ^{1}H NMR (500 MHz, $CDCl_3$) δ 8.08-8.05 (m, 2H), 7.18-7.15 (m, 2H), 4.44-4.41 (m, 1H), 4.05-4.02 (m, 1H), 3.89 (s, 2H), 3.44-3.38 (m, 1H), 3.32-3.26 (m, 1H), 3.11-3.05 (m, 1H), 2.41 (s, 3 H), 2.25-2.17 (m, 2H), 2.02-1.88 (m, 2H); ^{13}C NMR (500 MHz, $CDCl_3$) δ 180.66, 167.62, 165.71, 165.02, 163.70, 151.67, 149.85, 129.72, 129.65, 129.65, 116.25, 116.07, 45.27, 41.20, 34.05, 29.52, 28.91, 8.51; ^{19}F NMR (400 MHz, $CDCl_3$) δ -108.48 (S4 File).

1-((1R,5S,6S)-6-(3-(4-chlorophenyl)-1,2,4-oxadiazol-5-yl)-3-azabicyclo[3.1.1]heptan-3-yl)-2-(1-methyl-1H-1,2,4-triazol-5-yl)ethan-1-one (mCLF177)

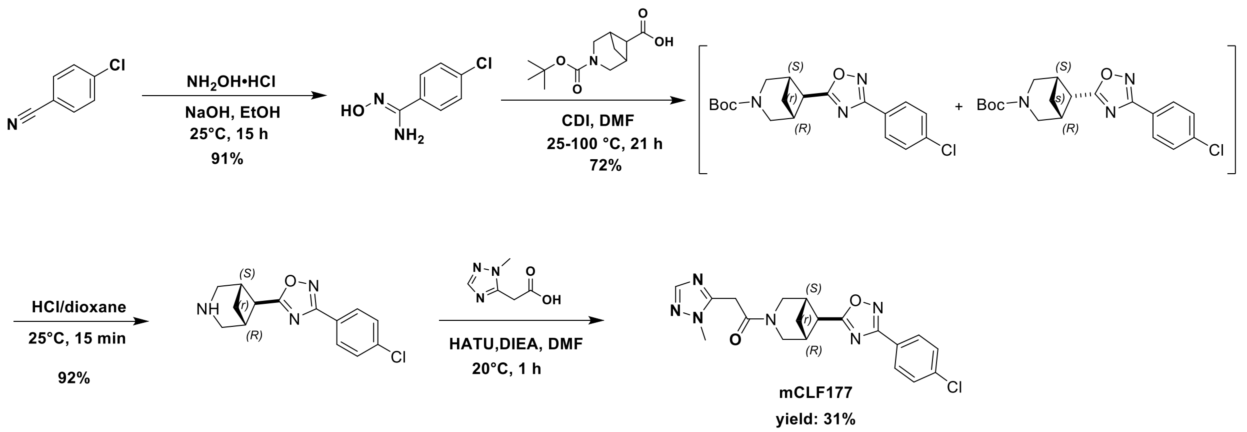

4-Chloro-N'-hydroxy-benzamidine

To a solution of 4-chlorobenzonitrile (12 g, 87 mmol, 1.0 *eq*) in EtOH (100 mL) was added NH_2OH•HCl (12.1 g, 174 mmol, 2.0 *eq*) and NaOH (7 g, 174 mmol, 2.0 *eq*) at 0°C. The mixture was stirred at 25 °C for 15 hours. TLC petroleum ether:ethyl acetate (1:1, v/v) showed 4-chlorobenzonitrile (Rf = 0.8) was consumed and a new large spot (Rf = 0.5) was formed. The mixture was poured into water (100 mL) and extracted with ethyl acetate (3 × 80 mL). Then the combined organic layer was washed with brine (80 mL), dried over Na_2SO_4, then the mixture was filtered and the filtrate was concentrated to afford 4-chloro-N'-hydroxy-benzamidine (15 g, 79.2 mmol, 91% yield) as a white solid. LC/MS [ESI, M+1]: m/z 170.9.

***Tert*-butyl (1R,5S,6r)-6-(3-(4-chlorophenyl)-1,2,4-oxadiazol-5-yl)-3-zabicyclo[3.1.1]heptane-3-carboxylate (*cis*)**

To a solution of 3-*tert*-butoxycarbonyl-3-azabicyclo [3.1.1]heptane-6-carboxylic acid (14.5 g, 60.1 mmol, 1.00 *eq*) in DMF (300 mL) was added CDI (9.74 g, 60.1 mmol, 1.00 *eq*). The mixture was stirred at 40 °C for 1 hour, then 4-chloro-N'-hydroxy-benzamidine (11.3 g, 66.1 mmol, 1.10 *eq*) was added. The mixture was stirred at 20°C for 2 hours, and then stirred at 100°C for another 21

hours. The reaction mixture was poured into H_2O (1.00 L) and extracted with ethyl acetate (2 × 0.5 L). The combined organic layers were washed with brine 500 mL, dried over Na_2SO_4, filtered and concentrated under reduced pressure to give a residue. The crude product was purified by silica gel column chromatography (petroleum ether: ethyl acetate = 50: 1 to 3: 1) to give *tert*-butyl (1R,5S,6r)-6-(3-(4-chlorophenyl)-1,2,4-oxadiazol-5-yl)-3-azabicyclo[3.1.1]heptane-3-carboxylate (*cis*, 11.5 g, 30.6 mmol, 50.9% yield) as white solid and *tert*-butyl (1R,5S,6s)-6-(3-(4-chlorophenyl)-1,2,4-oxadiazol-5-yl)-3-azabicyclo[3.1.1]heptane-3-carboxylate (*trans*, 10.8 g, 28.7 mmol, 47.8% yield) as white solid. LC/MS [ESI, M-55]: m/z 320.2.

5-((1R,5S,6r)-3-azabicyclo[3.1.1]heptan-6-yl)-3-(4-chlorophenyl)-1,2,4-oxadiazole (*cis*)

To a solution of *tert*-butyl (1R,5S,6r)-6-(3-(4-chlorophenyl)-1,2,4-oxadiazol-5-yl)-3-azabicyclo[3.1.1]heptane-3-carboxylate (12.5 g, 33.3 mmol, 1.0 *eq*) in DCM (65.0 mL) was added dropwise HCl/dioxane (4 M, 41.6 mL, 5.0 *eq*) at 20 °C. The reaction mixture was stirred at 20 °C for 12 hours. TLC petroleum ether:ethyl acetate (2:1, v/v), Rf = 0 showed the starting material was consumed completely. The reaction mixture was concentrated under reduced pressure to give a residue. The crude product was triturated with 2-methoxy-2-methylpropane (50 mL) at 20 °C for 5 mins. Then the mixture was filtered and the filter cake was concentrated under reduced pressure to give 5-((1R,5S,6r)-3-azabicyclo[3.1.1]heptan-6-yl)-3-(4-chlorophenyl)-1,2,4-oxadiazole (10.0 g, 96.3% yield, HCl) obtained as white solid. LC/MS [ESI, M+1]: m/z 276.1.

1-((1R,5S,6s)-6-(3-(4-chlorophenyl)-1,2,4-oxadiazol-5-yl)-3-azabicyclo[3.1.1]heptan-3-yl)-2-(1-methyl-1H-1,2,4-triazol-5-yl)ethan-1-one (mCLF177)

A mixture of 5-((1R,5S,6r)-3-azabicyclo[3.1.1]heptan-6-yl)-3-(4-chlorophenyl)-1,2,4-oxadiazole (2.86 g, 9.16 mmol, 1.0 eq, HCl), 2-(1-methyl-1H-1,2,4-triazol-5-yl)acetic acid (1.94 g, 13.7 mmol, 1.5 *eq*) and DIPEA (3.55 g, 27.5 mmol, 4.79 mL, 3.0 *eq*), EDCI (3.51 g, 18.3 mmol, 2.0 *eq*), HOBt (2.48 g, 18.3 mmol, 2.0 *eq*) in DCM (30 mL) was stirred at 20 °C for 2 hours. LC/MS showed the starting material was consumed completely and the desired compound was detected. The reaction mixture was poured into water (200 mL) and extracted with DCM (2 × 200 mL). The combined organic layer was washed with brine (200 mL), dried over Na2SO4, filtered and concentrated under reduced pressure to give a residue. The residue was purified by silica gel column chromatography (DCM: MeOH = 1: 0 to 100: 1) to yield crude product. The crude product was dissolved in DCM (100 mL), and 2-methoxy-2-methylpropane (10 mL) was added to the solution. Then, the mixture was filtered and the filter cake was concentrated under reduced pressure to give a product. The product was lyophilized to give 1-((1R,5S,6r)-6-(3-(4-chlorophenyl)-1,2,4-oxadiazol-5-yl)-3-azabicyclo[3.1.1]heptan-3-yl)-2-(1-methyl-1H-1,2,4-triazol-5-yl)ethan-1-one (*cis*, 3.6 g, 9.05 mmol, 98.8% yield) as white solid. LC/MS [ESI, M+1]: m/z 399.1. ^{1}H NMR (500 MHz, $CDCl_3$) δ 8.06 (s, 1H), 8.04 (d, *J* = 8.6 Hz, 2H), 7.46 (d, *J* = 8.6 Hz, 2H), 4.27 (s, 2H), 4.24-4.17 (m, 2H), 4.05 (s, 3 H), 3.88-3.87 (m, 2H), 3.22 (d, *J* = 5.8 Hz, 1H), 3.06-3.00 (m, 2H), 2.87-2.82 (m, 1H), 1.58 (dd, *J* = 6.0, 10.5 Hz, 1H); ^{13}C NMR (500 MHz, $CDCl_3$) δ 180.07, 167.82, 166.47, 149.23, 146.7, 137.53, 129.31 (2C), 128.84 (2C), 125.29, 51.95, 50.75, 41.26, 36.95, 36.53, 35.94, 31.16, 29.35 (S4 File).

1-(5-(3-(4-chloro-3-fluorophenyl)-1,2,4-oxadiazol-5-yl)-2-azabicyclo[2.2.1]heptan-2-yl)-2-(1-methyl-1H-1,2,4-triazol-5-yl)ethan-1-one (mCLF178)

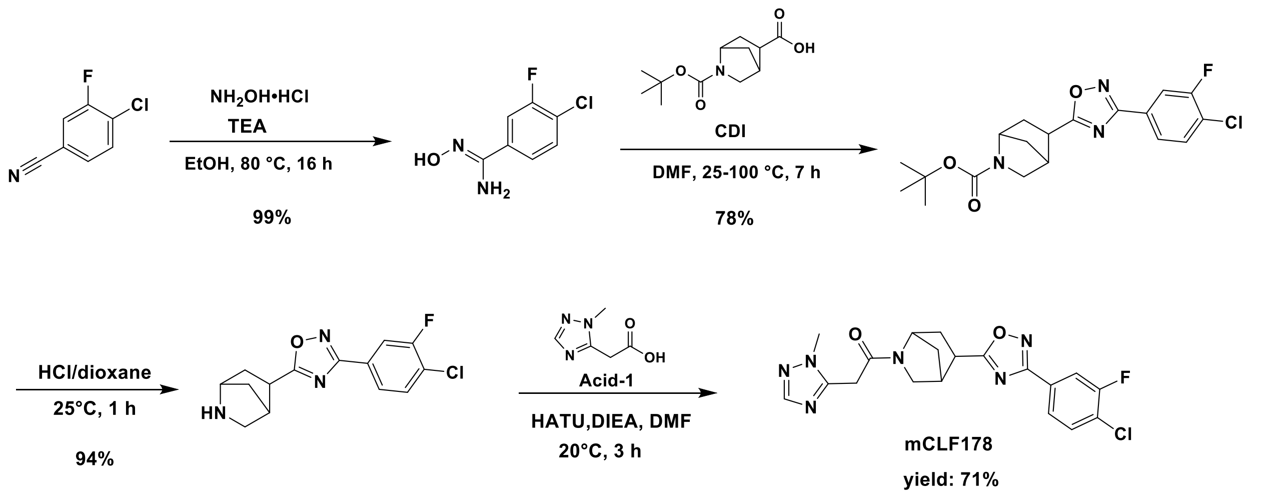

4-Chloro-3-fluoro-N'-hydroxybenzimidamide

To a solution of 4-chloro-3-fluoro-N'-hydroxybenzimidamide (15 g, 96.4 mmol, 1.0 *eq*) in EtOH (150 mL) was added NH_2OH•HCl (10 g, 145 mmol, 1.5 *eq*) and TEA (145 mmol, 20 mL, 1.5 *eq*), then the mixture was stirred at 80 °C for 16 hours. TLC petroleum ether:ethyl acetate (2:1, v/v) R_f = 0.71) showed 4-chloro-3-fluorobenzonitrile was consumed. The mixture was concentrated under vacuum and diluted with water (100 mL). The aqueous phase was extracted with ethyl acetate (3 × 50 mL). The combined organic phase was washed with brine (2 × 55 mL), dried over with Na_2SO_4, filtered and concentrated under vacuum to give 4-chloro-3-fluoro-N'-hydroxybenzimidamide (18.0 g, 74.7 mmol, 99% yield) as white solid. LC/MS [ESI, M+1]: m/z 189.1.

***Tert*-butyl 5-(3-(4-chloro-3-fluorophenyl)-1,2,4-oxadiazol-5-yl)-2-azabicyclo[2.2.1]heptane-2-carboxylate**

To a solution of 2-(*tert*-butoxycarbonyl)-2-azabicyclo[2.2.1]heptane-5-carboxylic acid (7.00 g, 29.0 mmol, 1.0 *eq*) in DMF (70.0 mL) was added CDI (5.60 g, 34.5 mmol, 1.2 *eq*) and the mixture was stirred at 25 °C for 1 hour, then added 4-chloro-3-fluoro-N'-hydroxybenzimidamide (8.40 g,

37.9 mmol, 1.3 *eq*) and the mixture was stirred at 100 °C for 6 hours. The mixture was concentrated under vacuum and diluted with water (100 mL). The aqueous phase was extracted with ethyl acetate (3 × 50 mL). The combined organic phase was washed with brine (2 × 55 mL), dried over with Na_2SO_4, filtered and concentrated under vacuum to give *tert*-butyl 5-(3-(4-chloro-3-fluorophenyl)-1,2,4-oxadiazol-5-yl)-2-azabicyclo[2.2.1]heptane-2-carboxylate (8.6 g, 22.6 mmol, 78% yield) as white solid. LC/MS [ESI, M-55]: m/z 338.1.

5-(2-Azabicyclo[2.2.1]heptan-5-yl)-3-(4-chloro-3-fluorophenyl)-1,2,4-oxadiazole

To a solution of tert-butyl 5-(3-(4-chloro-3-fluorophenyl)-1,2,4-oxadiazol-5-yl)-2-azabicyclo[2.2.1]heptane-2-carboxylate (8.00 g, 20.3 mmol, 1.0 *eq*) was added HCl/dioxane (4 M, 80 mL, 15.8 *eq*) and was stirred at 25 °C for 1 hour. TLC petroleum ether:ethyl acetate (2:1, v/v) showed starting material was consumed. The mixture was concentrated under vacuum to give 5-(2-azabicyclo[2.2.1]heptan-5-yl)-3-(4-chloro-3-fluorophenyl)-1,2,4-oxadiazole (HCl, 6.0 g, 21.3 mmol, 94% yield) as a white solid. LC/MS [ESI, M-55]: m/z 294.1.

1-(5-(3-(4-Chloro-3-fluorophenyl)-1,2,4-oxadiazol-5-yl)-2-azabicyclo[2.2.1]heptan-2-yl)-2-(1-methyl-1H-1,2,4-triazol-5-yl)ethan-1-one (mCLF178)

To a solution of 2-(2-methyl-1,2,4-triazol-3-yl)acetic acid (0.35 g, 2.48 mmol, 1.2 *eq*) in DMF (6 mL) was added HOBT (420 mg, 3.11 mmol, 1.5 *eq*) and EDCI (590 mg, 3.08 mmol, 1.5 *eq*), then added DIPEA (0.79 g, 6.11 mmol, 3.0 *eq*) and 5-(2-azabicyclo[2.2.1]heptan-5-yl)-3-(4-chloro-3-fluorophenyl)-1,2,4-oxadiazole (0.6 g, 2.04 mmol, 1.0 *eq*), the mixture was stirred at 25 °C for 12 hours. The mixture was concentrated under vacuum and diluted with water (10 mL) and extracted with ethyl acetate (3 × 5 mL). The combined organic phase was washed with brine (2 × 5 mL),

dried over with Na_2SO_4, filtered and concentrated under vacuum. The crude product was purified by reversed-phase HPLC (column: Phenomenex luna C18 150*40mm* 15um; mobile phase: [water(0.1%TFA)-ACN];B%: 30%-60%,10min) to give 1-(5-(3-(4-chloro-3-fluorophenyl)-1,2,4-oxadiazol-5-yl)-2-azabicyclo[2.2.1]heptan-2-yl)-2-(1-methyl-1H-1,2,4-triazol-5-yl)ethan-1-one (diastereomers, ratio 1:1, 0.6 g, 1.4 mmol, 71% yield) as a white solid. LC/MS [ESI, M+1]: m/z 417.1. ^{1}H NMR (500 MHz, $CDCl_3$) δ 8.05 (d, J = 26.7 Hz, 1 H), 7.85–7.78 (m, 2H), 7.75-7.48 (m, 1H), 4.74 (s, 1H), 4.19-4.07 (m, 2H), 4.04 (s, 3H), 4.02 (s, 3H), 3.76 (s, 1H), 3.48-3.42 (m, 1H), 3.35-3.28 (m, 1H), 2.39-2.21 (m, 2H), 2.02-1.77 (m, 2H); ^{13}C NMR (500 MHz, $CDCl_3$) δ 181.36, 181.16, 166.34, 162.38, 158.75, 156.76, 148.57, 146.44, 145.39 130.81, 130.79, 126.55, 123.72, 123.26, 115.22, 115.04, 57.97, 55.37, 52.94, 51.94, 42.43, 41.32, 37.38, 37.32, 37.13, 36.37, 36.23, 36.09, 34.87, 31.87, 30.86; ^{19}F NMR (500 MHz, $CDCl_3$) δ -113.92, -113.98 (S4 File).

1-(4-(3-(3,4-Dichlorophenyl)-1,2,4-oxadiazol-5-yl)piperidin-1-yl)-2-(1-methyl-1H-1,2,4-triazol-5-yl)ethan-1-one (mCLB073)

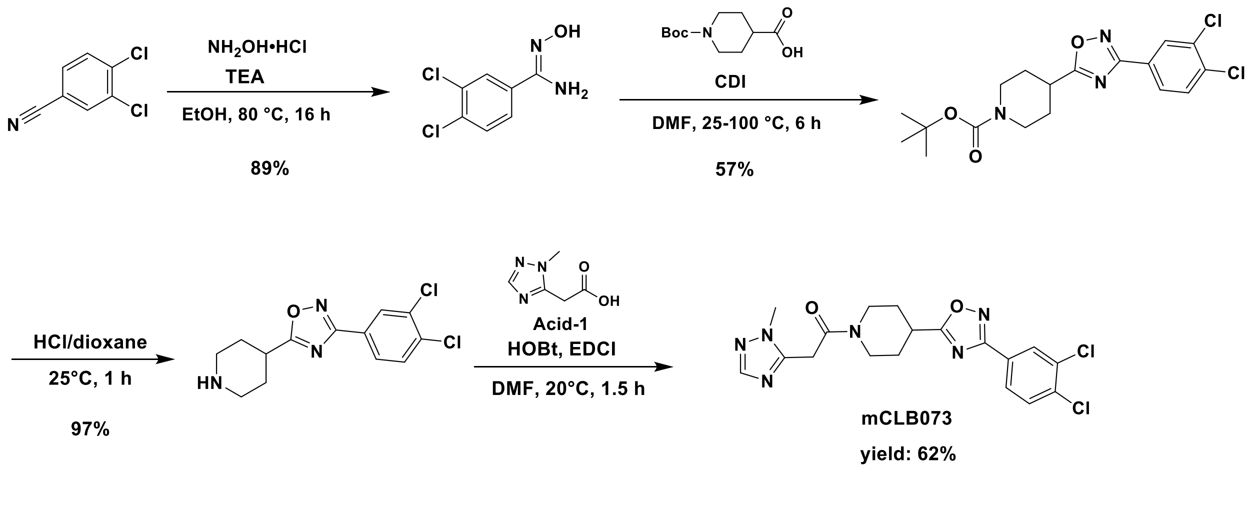

3,4-Dichloro-N'-hydroxybenzimidamide

To the mixture of 3,4-dichlorobenzonitrile (11.2 g, 65 mmol, 1.0 *eq*) in EtOH (100 mL) was added NH_2OH•HCl (4.9 g, 71.5 mmol, 1.1 *eq*) and NaOH (5.2 g, 130 mmol, 2.0 *eq*) at 0°C, then the mixture was stirred at 25 °C for 2 hours. TLC showed the reaction was complete. The mixture was poured into water (100 mL) and extracted with ethyl acetate (3 ×80 mL). Then the combined organic layer was washed with brine (80 mL), dried over Na_2SO_4, then the mixture was filtered and the filtrate was concentrated to afford 3,4-dichloro-N'-hydroxybenzimidamide (11.8 g, 57.8 mmol, 89% yield) as a white solid. LC/MS [ESI, M+1]: m/z 205.1.

***Tert*-butyl 4-(3-(3,4-dichlorophenyl)-1,2,4-oxadiazol-5-yl)piperidine-1-carboxylate**

To a solution of 1-(*tert*-butoxycarbonyl)piperidine-4-carboxylic acid (13.2 g, 57.8 mmol, 1.0 *eq*) in DMF (50 mL) was added CDI (9.4 g, 57.8 mmol, 1.0 *eq*). The mixture was stirred at 25 °C for 1 hour, then 3,4-dichloro-N'-hydroxybenzimidamide (11.8 g, 57.8 mmol, 1.0 *eq*) was added. The mixture was stirred at 25 °C for 2 hours, and then stirred at 100 °C for another 6 hours. The reaction mixture was poured into H_2O (100 mL) and extracted with ethyl acetate (3 × 100 mL). The combined organic phase was dried over Na_2SO_4, filtered and concentrated to afford *tert*-butyl 4-(3-(3,4-dichlorophenyl)-1,2,4-oxadiazol-5-yl)piperidine-1-carboxylate (18 g, 45.3 mmol, 78% yield) as white solid. LC/MS [ESI, M-55]: m/z 342.1.

3-(3,4-Dichlorophenyl)-5-(piperidin-4-yl)-1,2,4-oxadiazole

To a solution of 4-(3-(3,4-dichlorophenyl)-1,2,4-oxadiazol-5-yl)piperidine-1-carboxylate (18 g, 45.3 mmol, 1.00 *eq*) was added HCl/dioxane (4 M, 100 mL, 8.8 *eq*). The mixture was stirred at 20 °C for 1 hour. LC/MS showed 3-(3,4-dichlorophenyl)-5-(piperidin-4-yl)-1,2,4-oxadiazole was consumed. The mixture was filtered under vacuum to give 3-(3,4-dichlorophenyl)-5-(piperidin-4-

yl)-1,2,4-oxadiazole (HCl, 13.8 g, 46.5 mmol, 97% yield) as a white solid. LC/MS [ESI, M+1]: m/z 298.1.

1-(4-(3-(3,4-Dichlorophenyl)-1,2,4-oxadiazol-5-yl)piperidin-1-yl)-2-(1-methyl-1H-1,2,4-triazol-5-yl)ethan-1-one (mCLB073)

To a solution of 2-(1-methyl-1H-1,2,4-triazol-5-yl)acetic acid (4 g, 28.6 mmol, 1.0 *eq*, HCl) in DMF (20 mL) was added HOBT (5.9 g, 43.4 mmol, 1.52 *eq*) and EDCI (6.7 g, 43.2 mmol, 1.51 *eq*), then added DIPEA (11.1g, 85.8 mmol, 3.00 *eq*) and 3-(3,4-dichlorophenyl)-5-(piperidin-4-yl)-1,2,4-oxadiazole (8.5 g, 28.6 mmol, 1.0 *eq*). The mixture was stirred at 25 °C for 1.5 hours, and was concentrated under vacuum, then diluted with water (100 mL) and extracted with ethyl acetate (3 × 50 mL). The combined organic phase was washed with brine (2 × 50 mL), dried over with Na_2SO_4, filtered and concentrated under vacuum. The residue was purified by silica gel chromatography eluted with 0 - 50% ethyl acetate in hexane to afford 1-(4-(3-(3,4-dichlorophenyl)-1,2,4-oxadiazol-5-yl)piperidin-1-yl)-2-(1-methyl-1H-1,2,4-triazol-5-yl)ethan-1-one (7.4 g, 17.6 mmol, 62% yield) as white solid. LC/MS [ESI, M+1]: 421.1. ^{1}H NMR (500 MHz, $CDCl_3$) δ 8.16 (d, J = 3.0 Hz, 1H), 8.02 (s, 1H), 7.89 (dd, J = 2.0, 8.2 Hz, 1H), 7.55 (d, J = 8.3 Hz, 1H), 4.37 (dt, J = 4.0, 13.3 Hz, 1H), 4.26-4.17 (m, 3H), 3.99 (s, 3H), 3.48-3.43 (m, 1H), 3.32-3.26 (m, 1H), 3.13-3.07 (m, 1H), 2.28-2.24 (m, 1H), 2.21-2.16 (m, 1H), 2.02-1.87 (m, 2H); ^{13}C NMR (500 MHz, $CDCl_3$) δ 18.55, 166.24, 163.27, 148.76, 146.24, 135.13, 132.90, 130.55, 128.85, 126.10, 126.00, 45.02, 40.49, 36.19, 33.43, 30.83, 28.95, 28.18 (S4 File).
