# Supplementary material for: Pharmacological and genetic activation of cAMP synthesis disrupts cholesterol utilization in Mycobacterium tuberculosis
Source: PLoS Pathog. 2022 Feb 8;18(2):e1009862. doi: 10.1371/journal.ppat.1009862 (PMC8856561; doi:10.1371/journal.ppat.1009862)

V-59 ^{1}H NMR

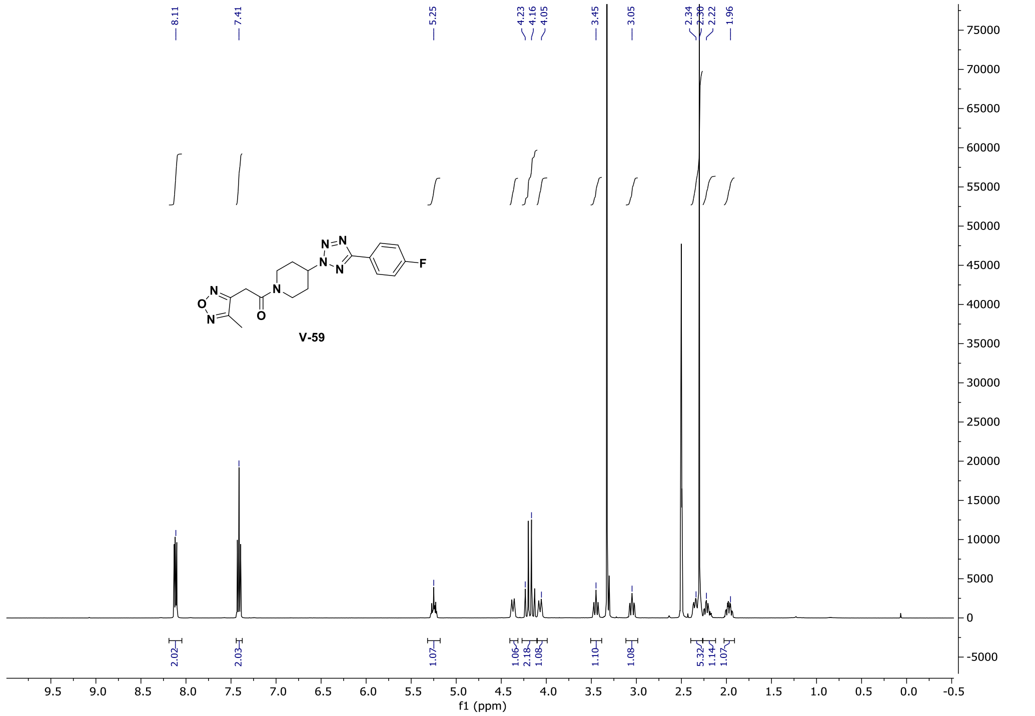

V-59 ^{13}C NMR

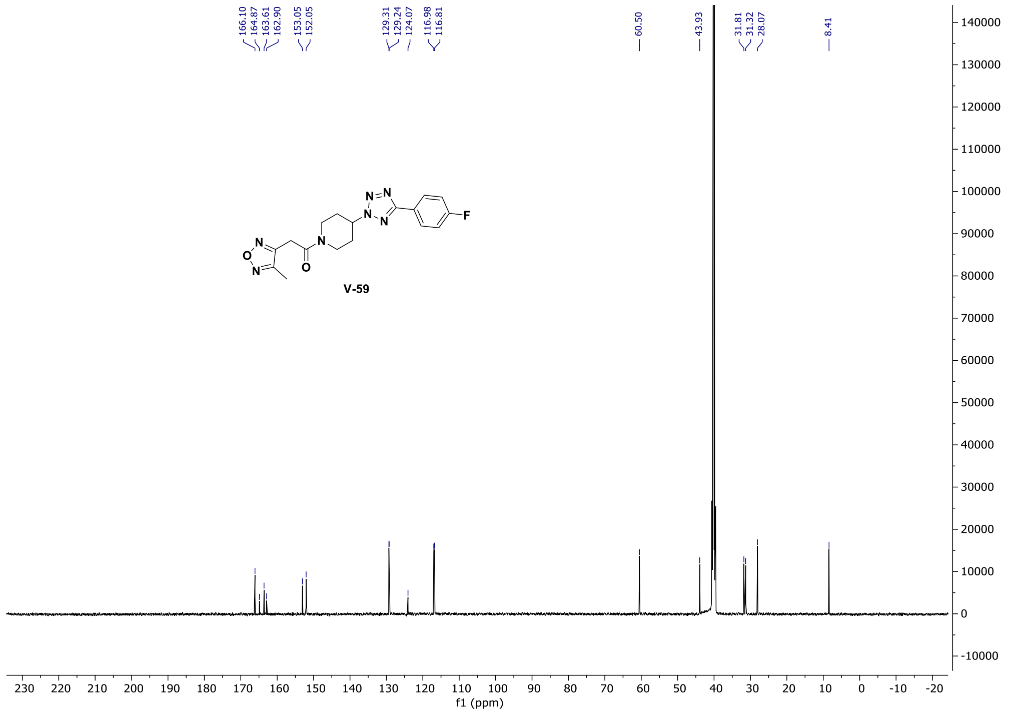

V-59 ^{19}F NMR

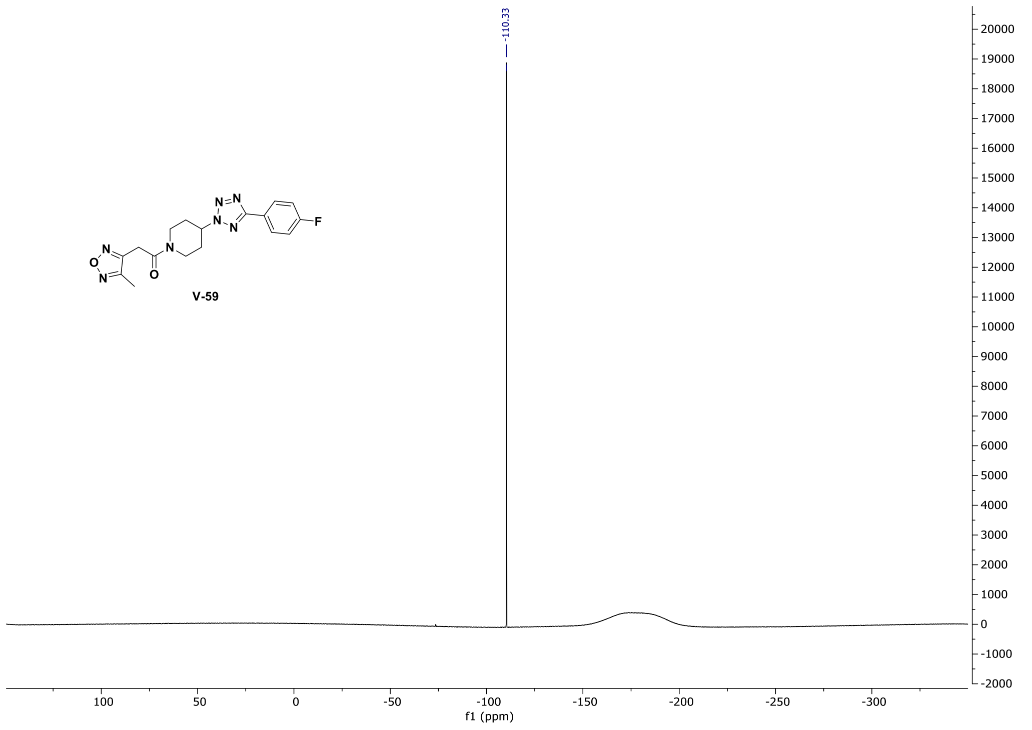

mCIS635 ^{1}H NMR

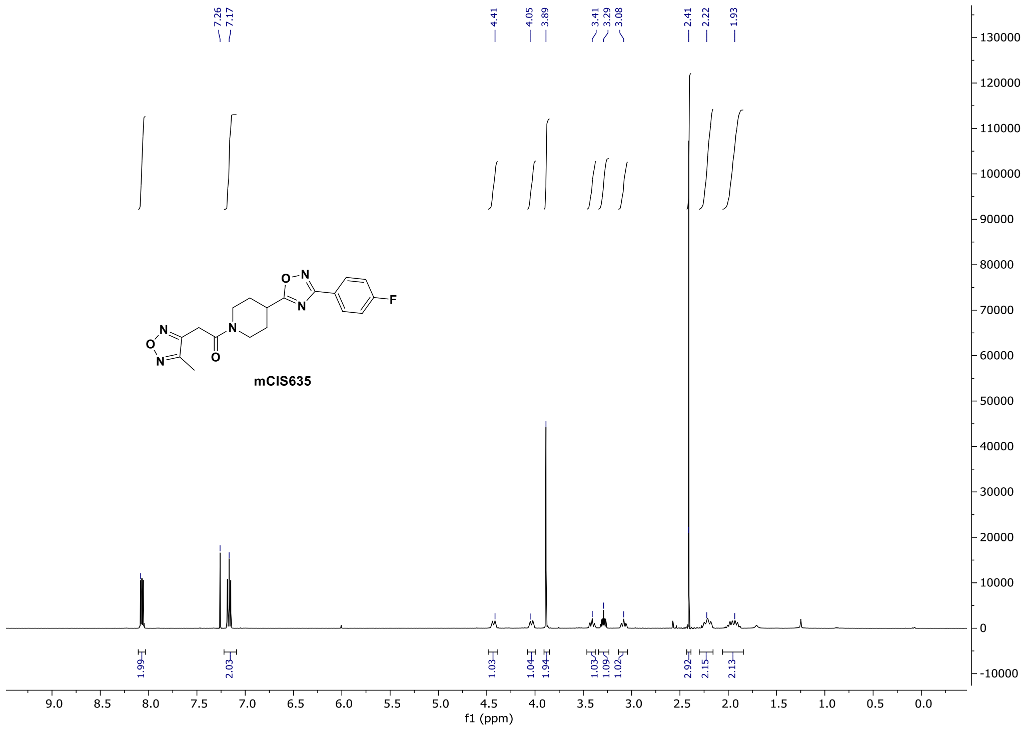

mCIS635 ^{13}C NMR

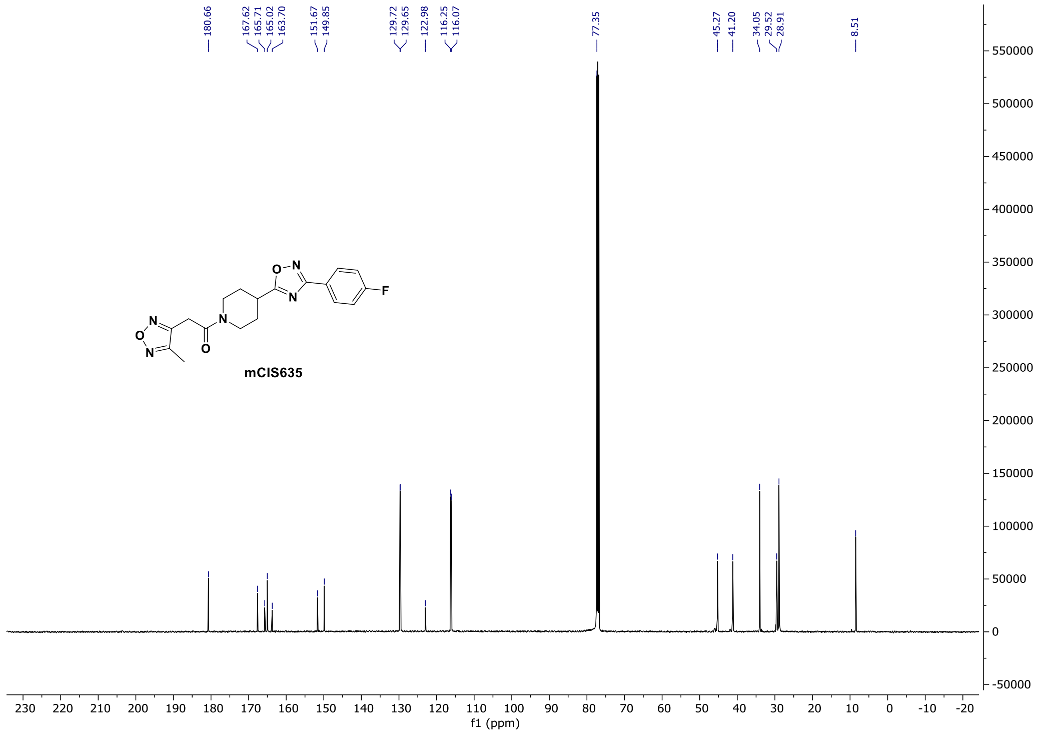

mCIS635 ^{19}F NMR

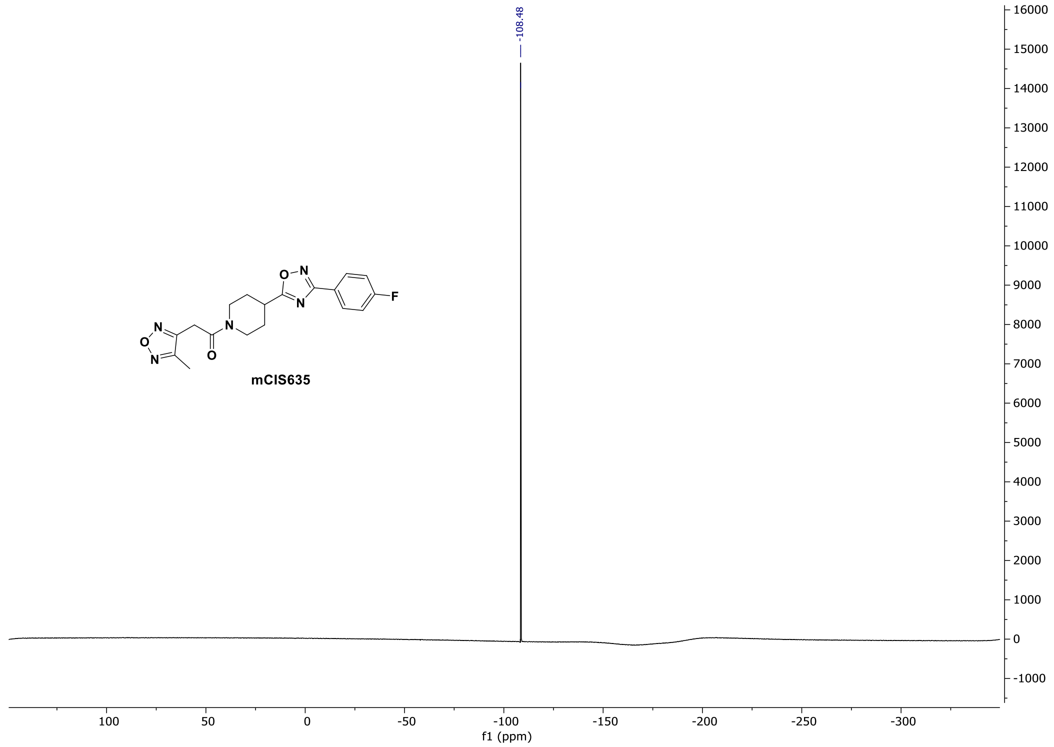

mCLE299 ^{1}H NMR

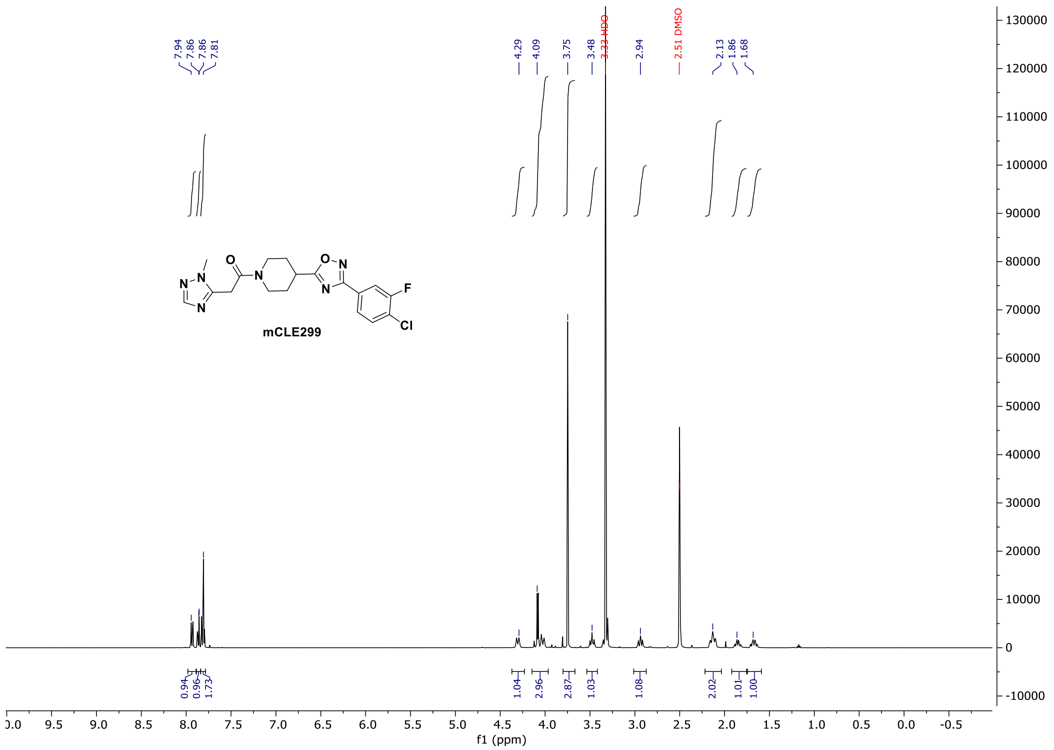

mCLE299 ^{13}C NMR

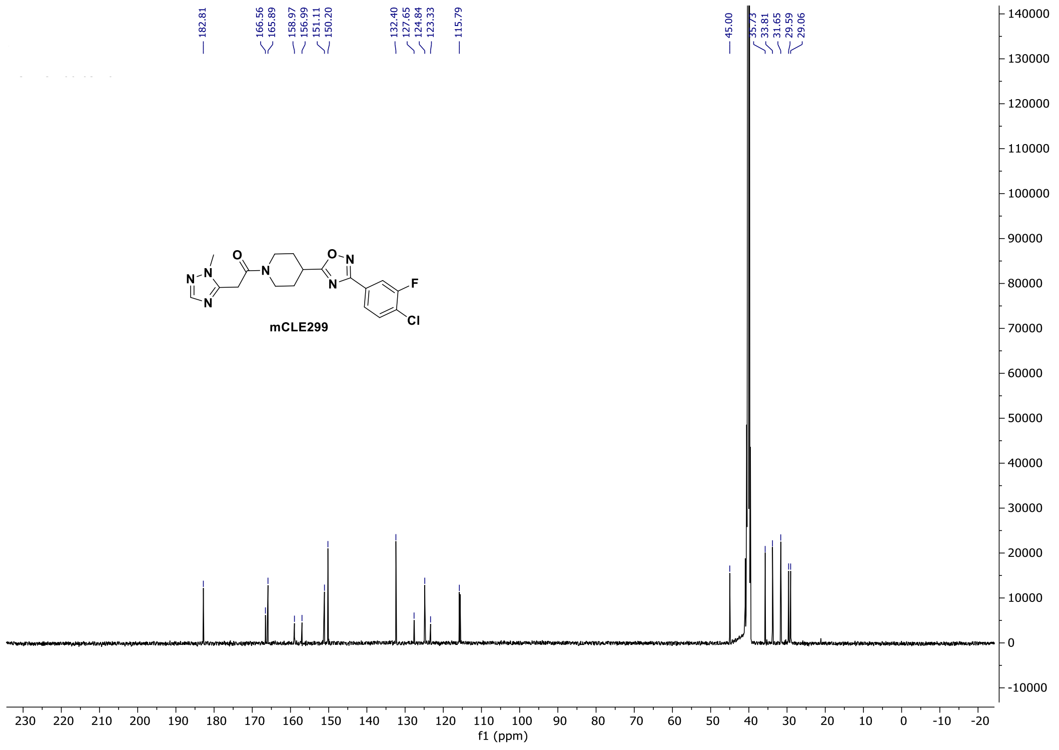

mCLE299 ^{19}F NMR

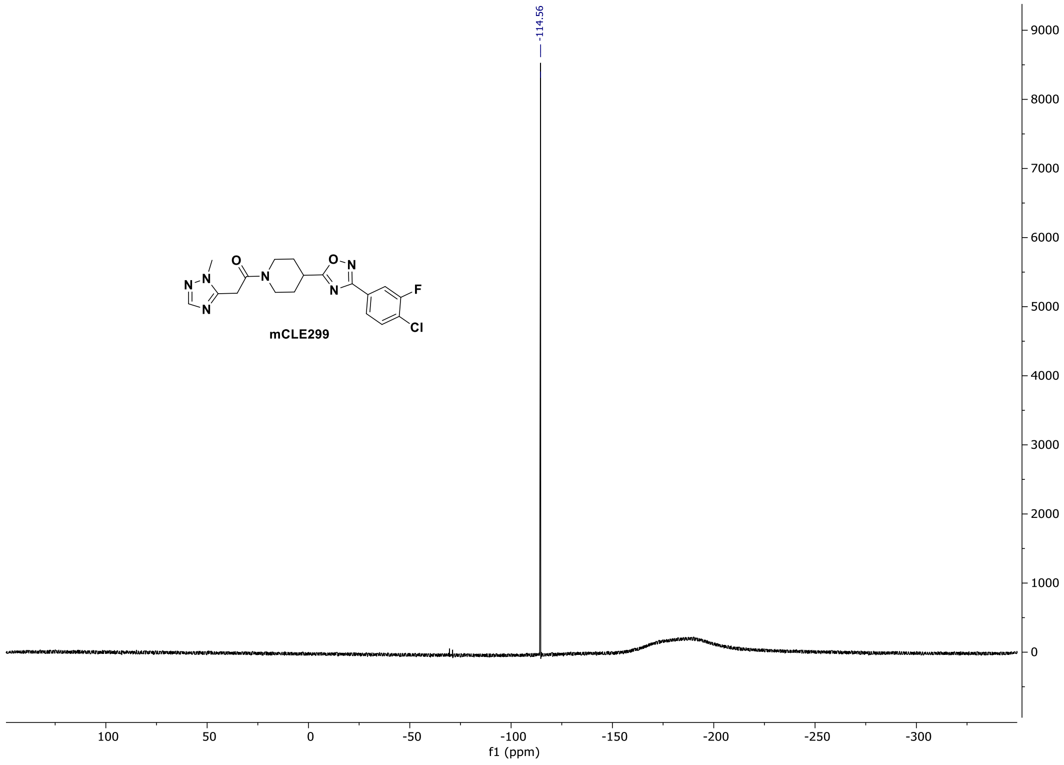

mCLF177 ^{1}H NMR

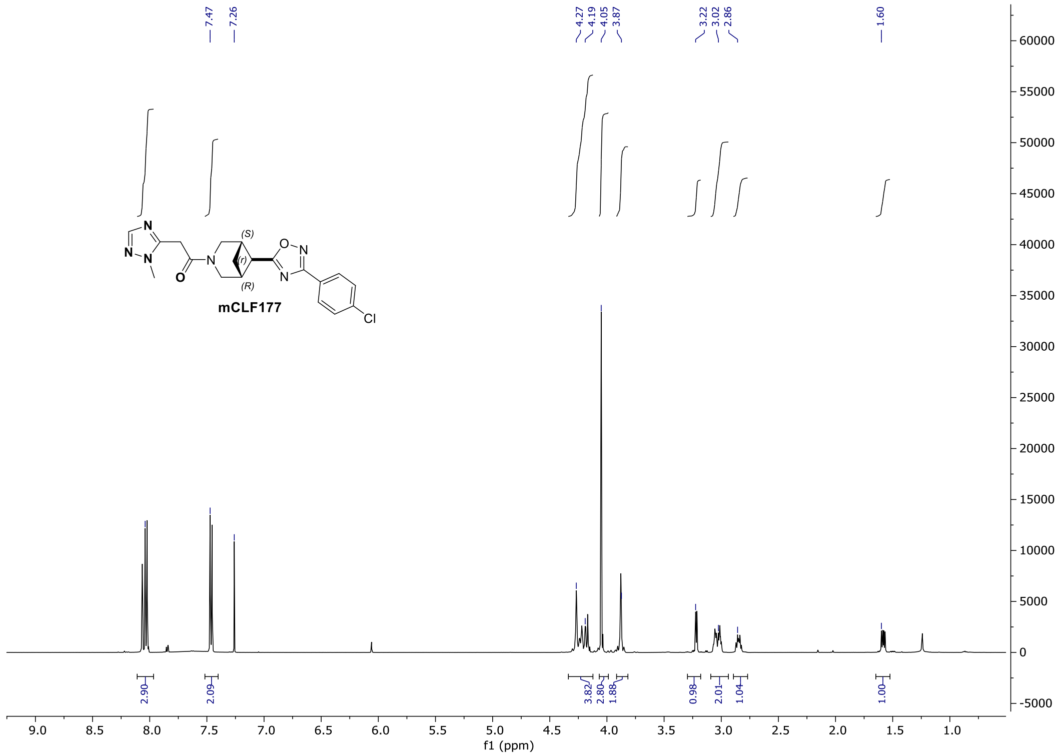

mCLF177 ^{13}C NMR

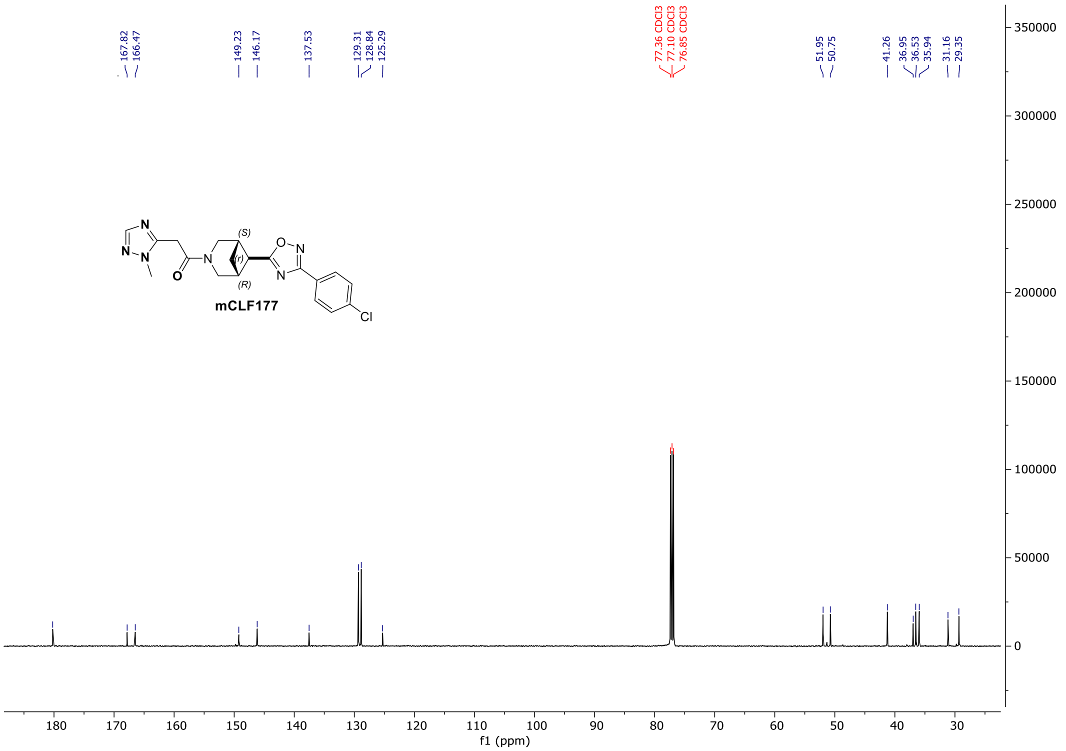

mCLF178 ^{1}H NMR

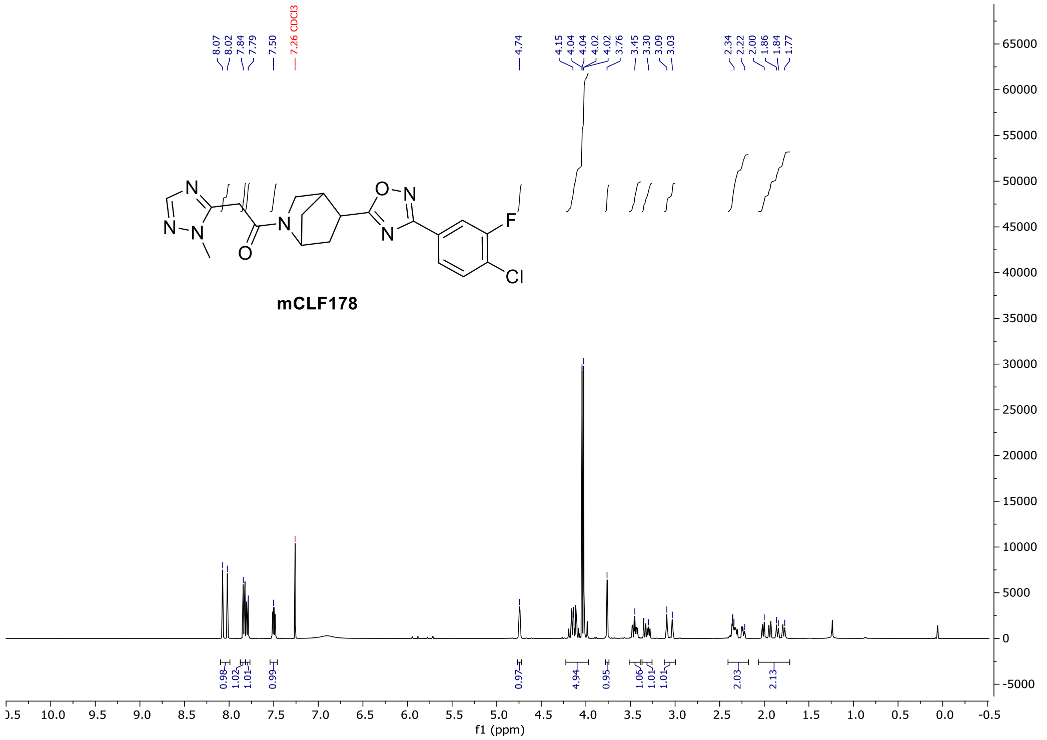

mCLF178 ^{13}C NMR

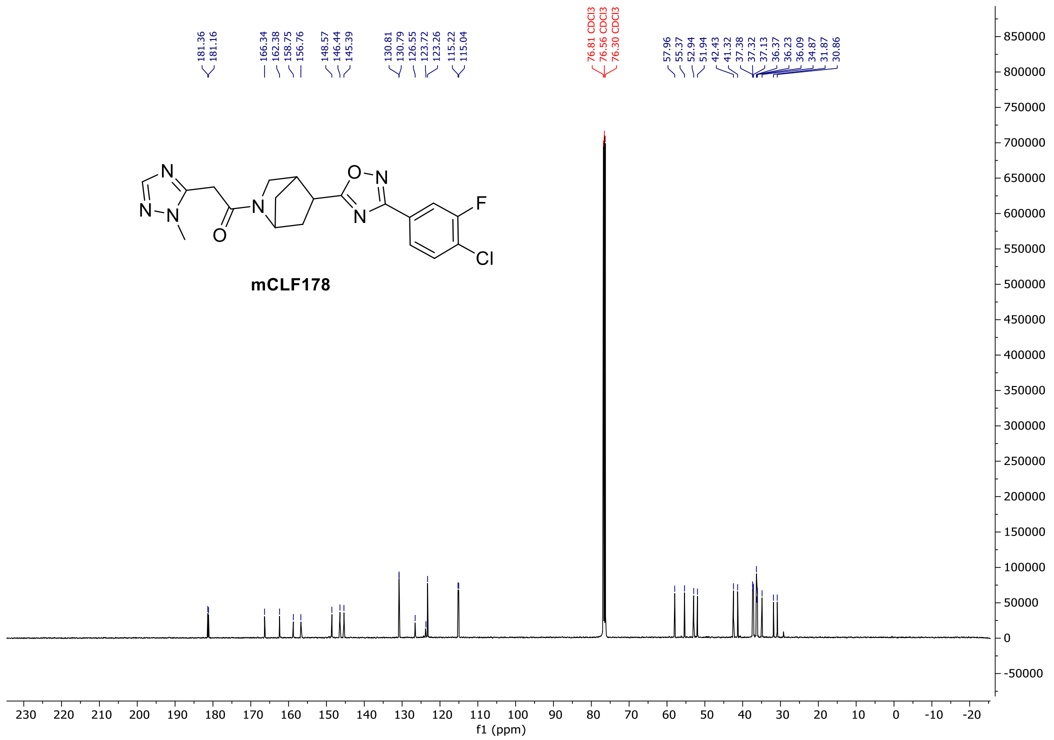

mCLF178 ^{19}F NMR

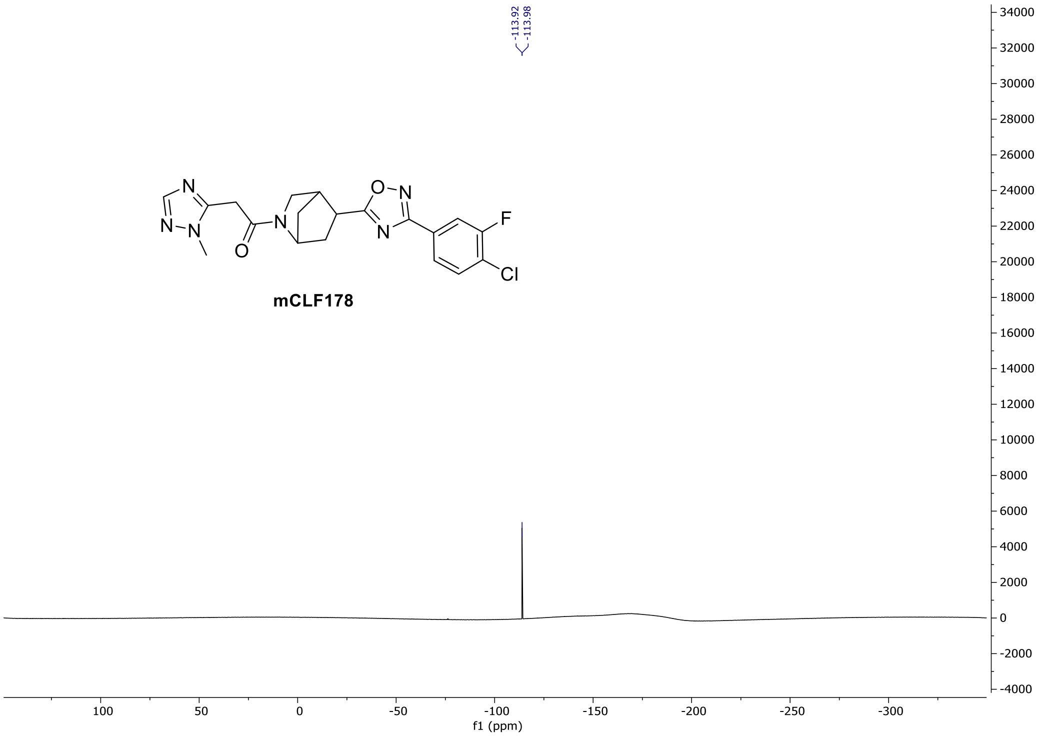

mCLB073 ^{1}H NMR

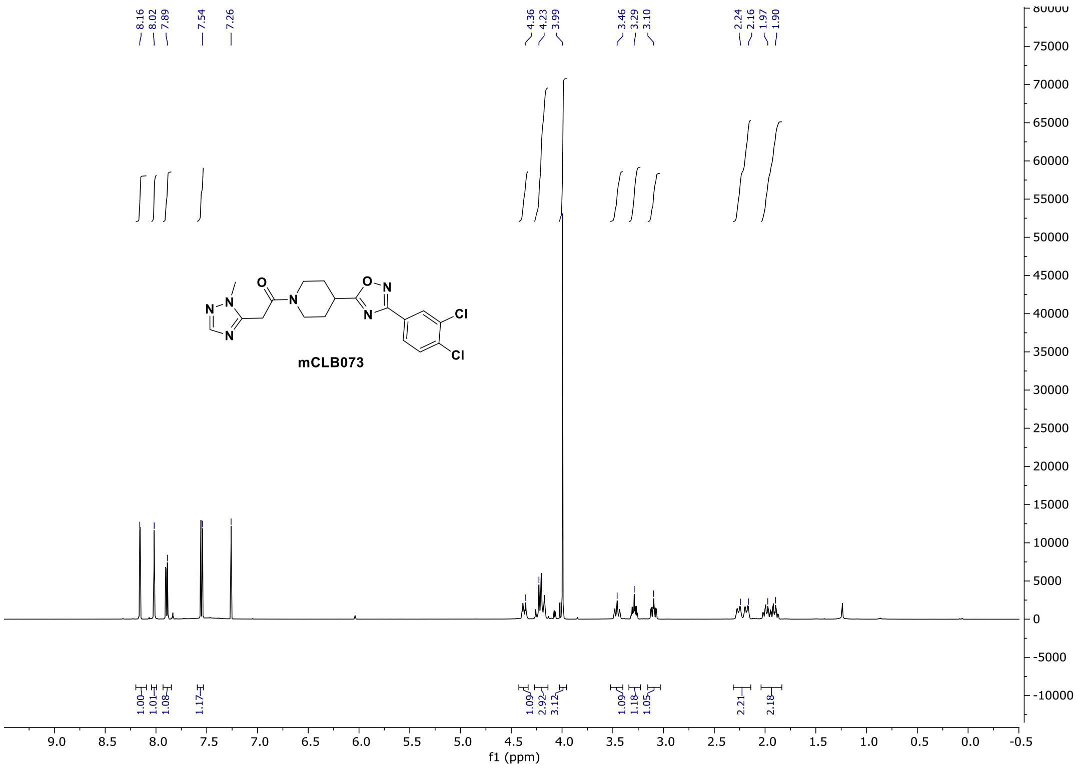

mCLB073 ^{13}C NMR

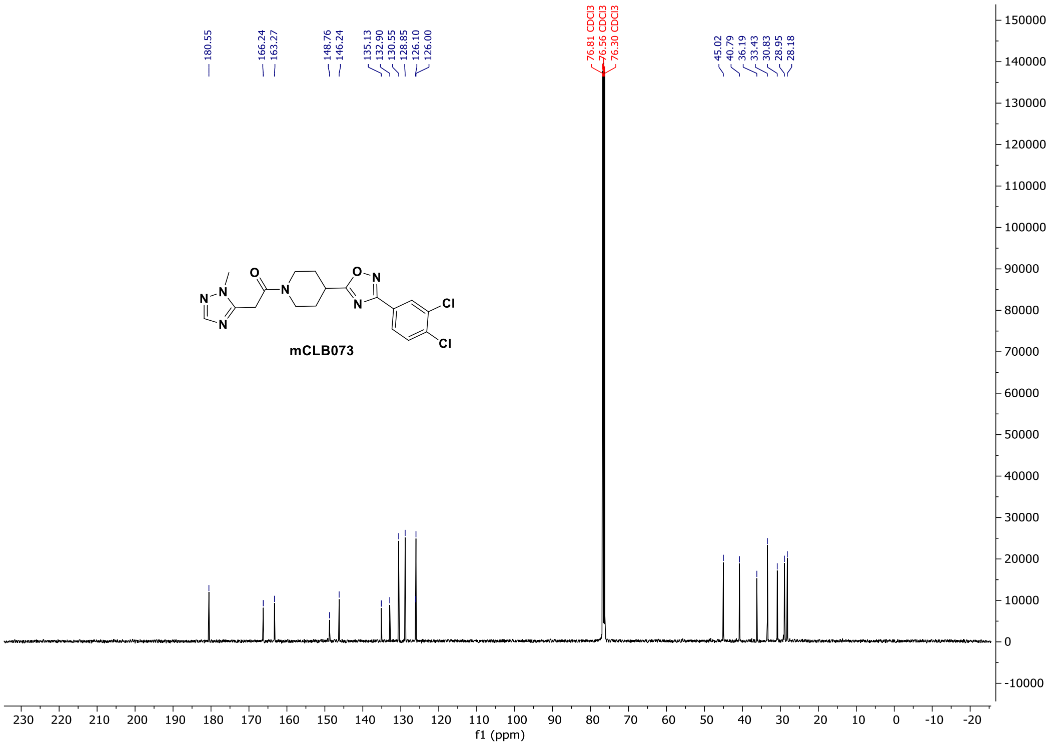

mCLF024 ^{1}H NMR

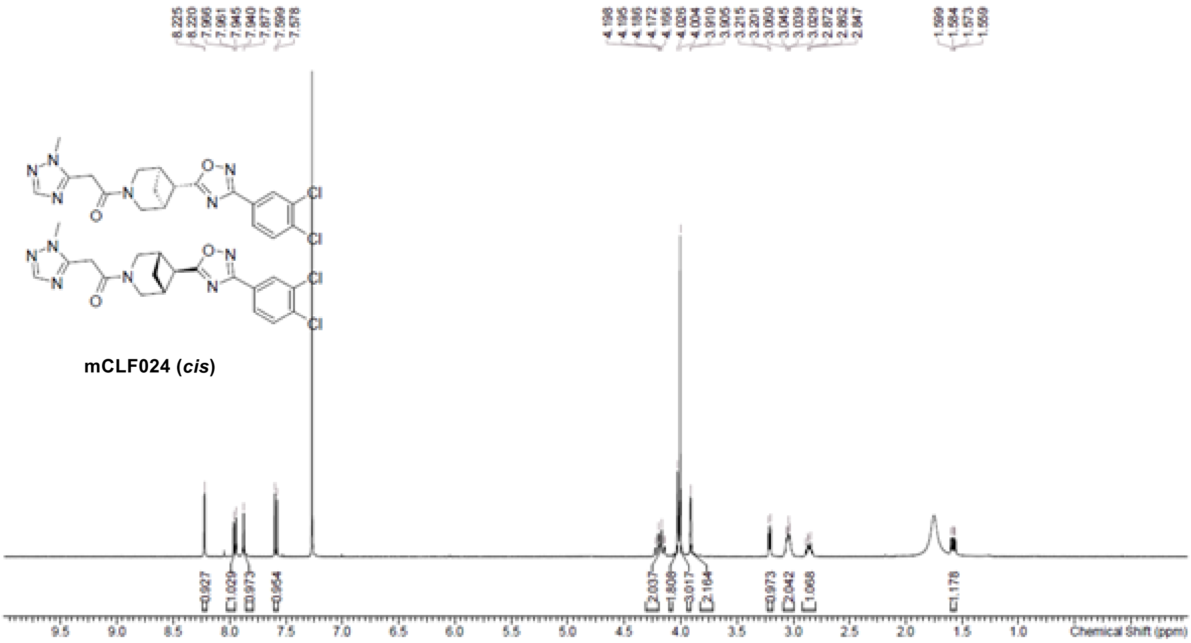

mCLF025 ^{1}H NMR

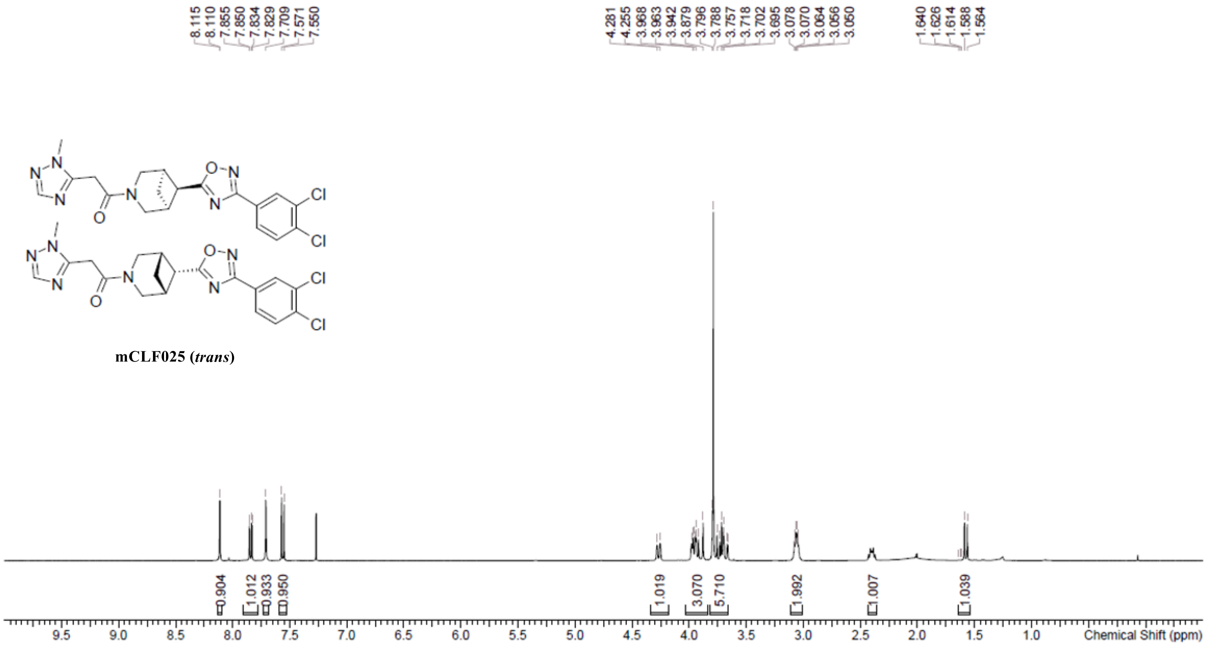

mCLF025 ^{13}C NMR

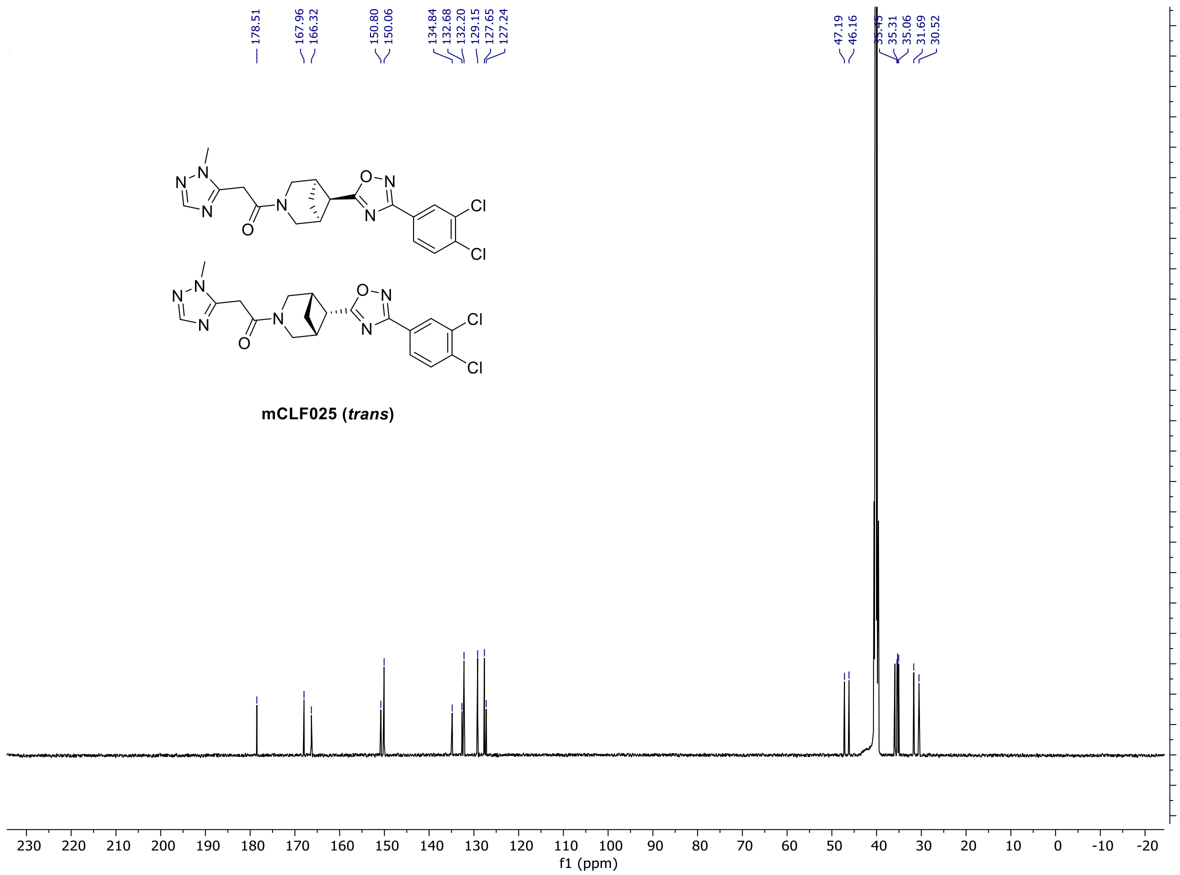

Supplement: S4 File — (PDF) [file ppat.1009862.s012.pdf]
